# Intrathecal Drug Delivery: Advances and Applications in the Management of Chronic Pain Patient

**DOI:** 10.3389/fpain.2022.900566

**Published:** 2022-06-16

**Authors:** Jose De Andres, Salim Hayek, Christophe Perruchoud, Melinda M. Lawrence, Miguel Angel Reina, Carmen De Andres-Serrano, Ruben Rubio-Haro, Mathew Hunt, Tony L. Yaksh

**Affiliations:** ^1^Surgical Specialties Department, Valencia University Medical School, Valencia, Spain; ^2^Anesthesia Critical Care and Pain Management Department, Valencia, Spain; ^3^Department of Anesthesiology, University Hospitals Cleveland Medical Center, Cleveland, OH, United States; ^4^Pain Center and Department of Anesthesia, La Tour Hospital, Geneva, Switzerland; ^5^Lausanne University Hospital and University of Lausanne, Lausanne, Switzerland; ^6^Department of Anesthesiology, Montepríncipe University Hospital, Madrid, Spain; ^7^CEU-San-Pablo University School of Medicine, Madrid, Spain; ^8^Department of Anesthesiology, University of Florida College of Medicine, Gainesville, FL, United States; ^9^Facultad de Ciencias de la Salud Universidad Francisco de Vitoria, Madrid, Spain; ^10^Multidisciplinary Pain Clinic, Vithas Virgen del Consuelo Hospital, Valencia, Spain; ^11^Anesthesia and Pain Management Department, Provincial Hospital, Castellon, Spain; ^12^Multidisciplinary Pain Clinic, Vithas Virgen del Consuelo Hospital, Valencia, Spain; ^13^Department of Physiology, Karolinska Institute, Stockholm, Sweden; ^14^Departments of Anesthesiology and Pharmacology, University of California, San Diego, San Diego, CA, United States

**Keywords:** antisense, intrathecal, neuromodulation, chronic pain, implantable drug delivery system (IDDS)

## Abstract

Advances in our understanding of the biology of spinal systems in organizing and defining the content of exteroceptive information upon which higher centers define the state of the organism and its role in the regulation of somatic and automatic output, defining the motor response of the organism, along with the unique biology and spatial organization of this space, have resulted in an increased focus on therapeutics targeted at this extracranial neuraxial space. Intrathecal (IT) drug delivery systems (IDDS) are well-established as an effective therapeutic approach to patients with chronic non-malignant or malignant pain and as a tool for management of patients with severe spasticity and to deliver therapeutics that address a myriad of spinal pathologies. The risk to benefit ratio of IDD makes it a useful interventional approach. While not without risks, this approach has a significant therapeutic safety margin when employed using drugs with a validated safety profile and by skilled practioners. The present review addresses current advances in our understanding of the biology and dynamics of the intrathecal space, therapeutic platforms, novel therapeutics, delivery technology, issues of safety and rational implementation of its therapy, with a particular emphasis upon the management of pain.

## Overview

Delivery of therapeutics to the spinal cord may be appropriate for three reasons. (1) The therapeutic targets are associated with spinal systems: dorsal root ganglion cell, nerve root, dorsal or ventral horn or the intrathecal space itself (meninges). Many states of pain and altered motor function represent changes in normal spinal function induced by peripheral tissue and nerve injury or by changes secondary to spinal trauma (section, ischemia or compression) or neurodegenerative processes (amyotrophic lateral sclerosis, somatomotor atrophy). Pathological processes may occur, such as cancer (meningeal carcinomatosis, chordoma) or bacterial/fungal infection (meningitis) that involve the intrathecal space and its contents. (2) Spinal delivery of the therapeutic may be required because the therapeutic platform does not have systemic access to the spinal space. Such examples would be large molecules (such as ziconotide and growth factors), antibodies, viral transfection platforms delivering siRNA/shRNA or oligonucleotides, which have restricted CNS access because of efficient blood brain barrier function. (3) The therapeutic agent with a spinal target may be effective after systemic delivery, but delivery of the agent directly into the spinal canal permits reduces systemic drug exposure while allowing high target concentrations with fewer effects on peripheral and non-spinal systems. Such conditions may serve to optimize therapeutic results by reducing the side effect profile. As discussed in this document, this foundation has been widely demonstrated with molecules, such as opiates or baclofen; although they are systemically effective in their intrathecal administration for the treatment of pain and spasticity, they reduce systemic exposure, with a reduction in secondary effects and improvement of the outcome.

Importantly, substantial advances in our understanding of neuraxial biology have revealed a myriad of novel targets in the dorsal horn and the dorsal root ganglia that regulate nociceptive processing. Particularly exciting is the evolving implementation by the neuraxial route of novel therapeutic platforms, such as toxins and gene targeting to interdict nociceptive processing by intrathecal delivery. This focus has resulted in an increased interest in the fluid dynamics of the extracranial neuraxial space and approaches to target more reliably the distribution requirements of the different pathologies. Thus, some indications may require limited segmental effects (as with various pain or spasticity indications), while others may require a broader distribution (as with meningeal cancers or neurodegenerative disorders). Here, the use of patient specific infusion protocols has become a point of interest, and we see changes in pump delivery profiles (programming), catheter construction, and configurations to allow broader distributions of small volumes of infusate evenly along the extent of the neuraxis and reduce the risk of local concentration-dependent pathologies that may arise due to the restricted redistribution of infusate, which may occur with small volume neuraxial infusion into a relatively low flow space. In the following sections, we address these evolving issues, which currently impact advances in the utilization of the neuraxial route of delivery.

## New Insights Into Extracranial Neuraxial Anatomy

The dural sac is the key structure for the distribution of infused drugs at the subarachnoid level. The spinal dural sac, within which is the cerebrospinal fluid, is constituted of the outer layer of the dura and the closely adherent inner arachnoid layer, forming the outer barrier of the spinal subarachnoid space (SSAS). The spinal margins of the SSAS are formed by the pia, which in itself is intimately associated with the surface of the spinal parenchyma and surrounds the cord in all aspects. Interior to the pia mater is the found glial limitans and beyond the glia and neurons. The pial layer of the spinal meninges represents the main barrier governing the transfer of drugs between the CSF and the spinal cord (1–4). The structural complexity of SSAS comprises the trabecular connection between the arachnoid and pia mater, the subarachnoid ligaments that periodically attach the space in a discontinuous manner along the longitudinal axis, and the nerve roots that emerge from the dorsal and ventral horns.

### Dura Mater

**Figure 1:**
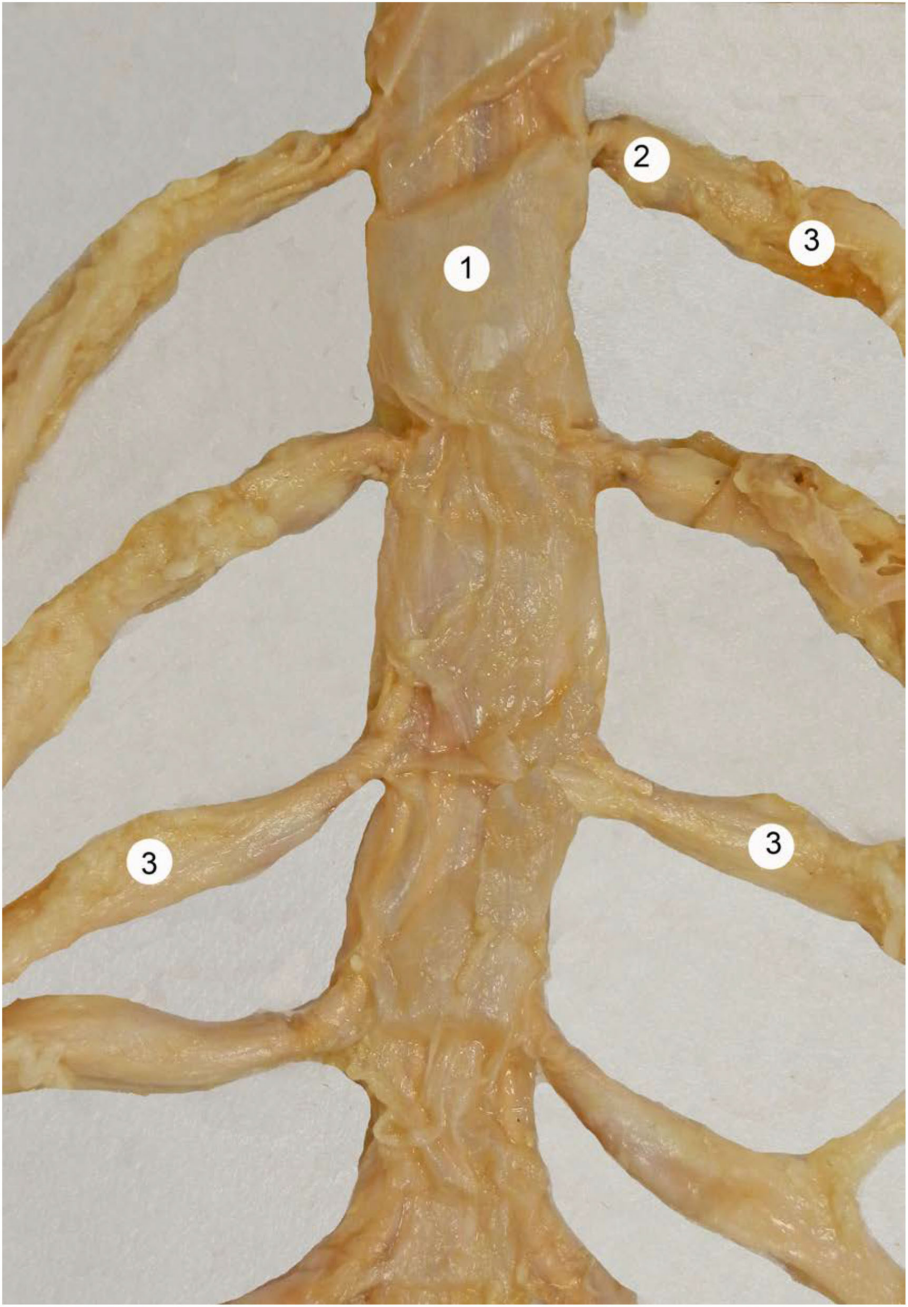
Human dural sac at the lumbar region. 1 = dural sac, 2 = nerve root cuff, 3 = dorsal root ganglia. With permission of Dr. Miguel A. Reina.

**Figure 2:**
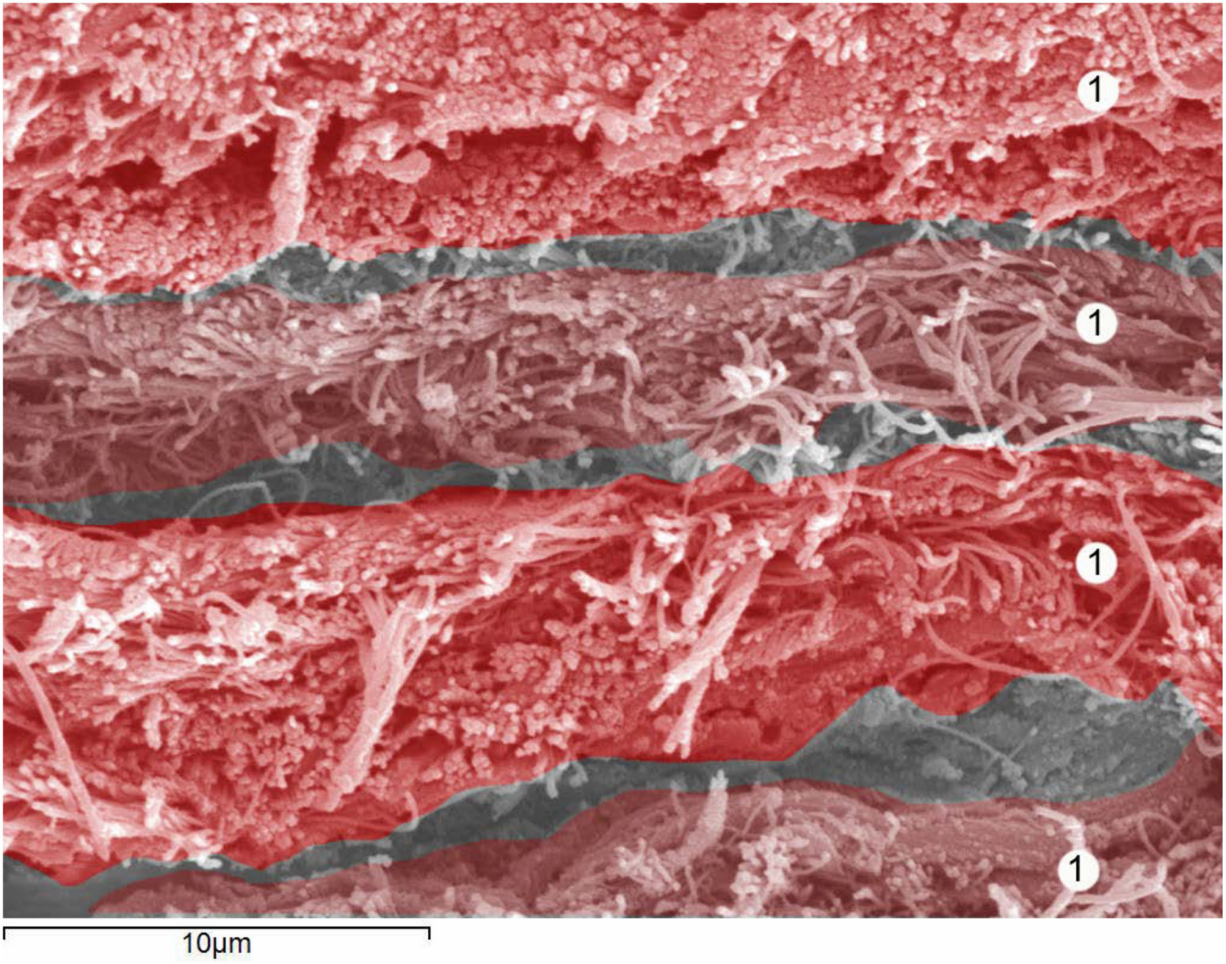
Human dura mater. Details of 4 dural lamina. Scanning electron microscopy. Magnification × 4,000. 1 = Dural lamina. With permission of Dr. Miguel A. Reina.

As shown in Figure 1, the dura mater, the most external layer, represents 90% of the total thickness of the dural sac. This fibrous structure, permeable to small molecules, confers significant mechanical resilience to the dural sac. The dura mater has a thickness of about 0.35 millimeters (mm) (0.25–0.40) (5). It is comprised of around 80 concentric dural laminas composed of collagen fibers distributed at random in all spatial directions, forming a touch semi-permeable matrix through which small, low molecular weight products can pass (e.g., the route of drug movement after epidural delivery) (Figure 2).

### Arachnoid

**Figure 3:**
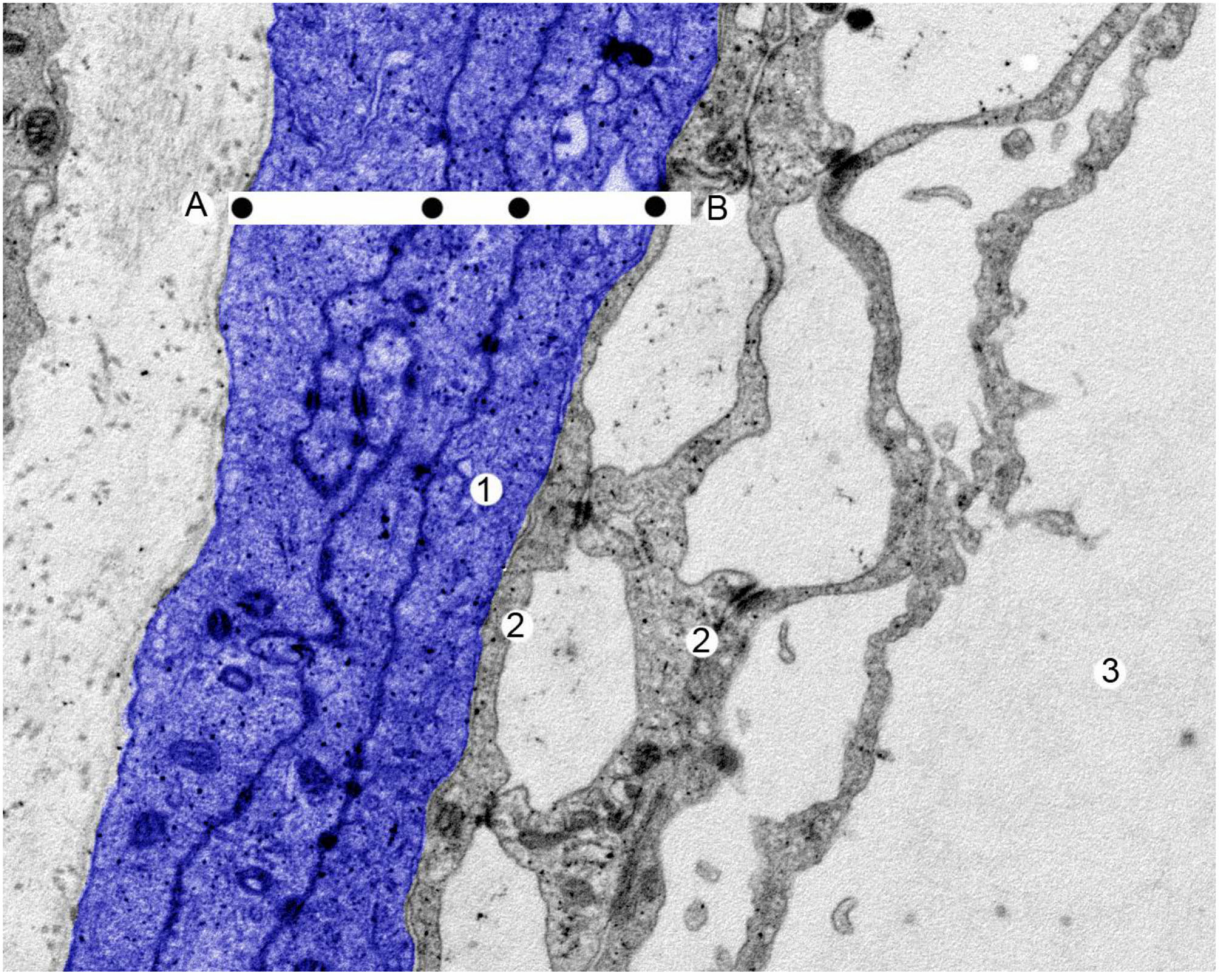
A human arachnoid layer. Scanning electron microscopy. Magnification x 25,000. The entire thickness of the arachnoid layer in blue. 1 = arachnoid cell, 2 = arachnoid trabeculate, 3 = cerebrospinal fluid. The drugs diffuse through the arachnoid layer from **(A)** to **(B)**. The black dots mark the plasmatic membrane of arachnoid cells where the molecules are lipid resistant. With permission of Dr. Miguel A. Reina.

The remaining internal 10% of the dural sac is formed by the arachnoid layer, which is a semi-permeable cellular layer, governing the passage of substances through the dural sac. The arachnoid layer has a thickness of 50–60 microns (μm) (Figure 3). Its barrier properties are due to arachnoid cells strongly bonded by specific plasmatic membrane junctions, such as tight junctions and desmosomes. There is no intercellular space between the cells, and molecules administered within the epidural space pass through the cell-rich subarachnoid layer. Accordingly, arachnoid passage is increased by low molecular weight solute, its lipophilicity, and reduced by hydrophobicity (6) (Figure 3). Although the arachnoid is essentially avascular, it provides a barrier protection for products moving from the fenestrated dural vessels. The outer most layer acts as a size-selective barrier through expression of claudin-11 in tight junctions to regulate movement of dural vessel-derived components (7).

The arachnoid layer and the underlying pial membrane to be discussed below are linked by several components. The trabecular arachnoid, bridging the arachnoid layer to the pia mater, surrounds nerve roots and free blood vessels that pass though the SSAS, providing to each one an arachnoid sleeve. The thickness of an arachnoid sleeve ranges from 10 to 60 μm. In some cases, one or more nerve roots are enclosed by a single arachnoid sleeve, and, in others, the nerve root has no arachnoid sleeves at all (1, 2). Trabecular arachnoid and subarachnoid ligaments anchor the lateral, anterior, and posterior sides of the spinal cord to the dural sac (8). These dentate ligaments are reliably present and serve to secure each side of the spinal cord to the dural sac. They serve as a land mark, demarcating the dorsal vs. ventral quadrants of the spinal cord. Less commonly, posterior ligaments (posticum) are formed of thin, inconsistent bands that attach the posterior spinal cord to the inner surface of the dural sac (5). Both posterior and posterior-lateral ligaments extend longitudinally from the cervical to the mid thoracic or lumbar level. The thinner ventral ligament is found on the anterior side of the SSAS. These subarachnoid ligaments do not limit fluid movement, but serve as impediments to linear flow patterns in the CSF and serve to locally disrupt the flow leading to increased local turbulence as driven by the local oscillatory movement, which is discussed further below (9), and the movement of an intrathecal therapeutic.

#### Pia Mater

**Figure 4:**
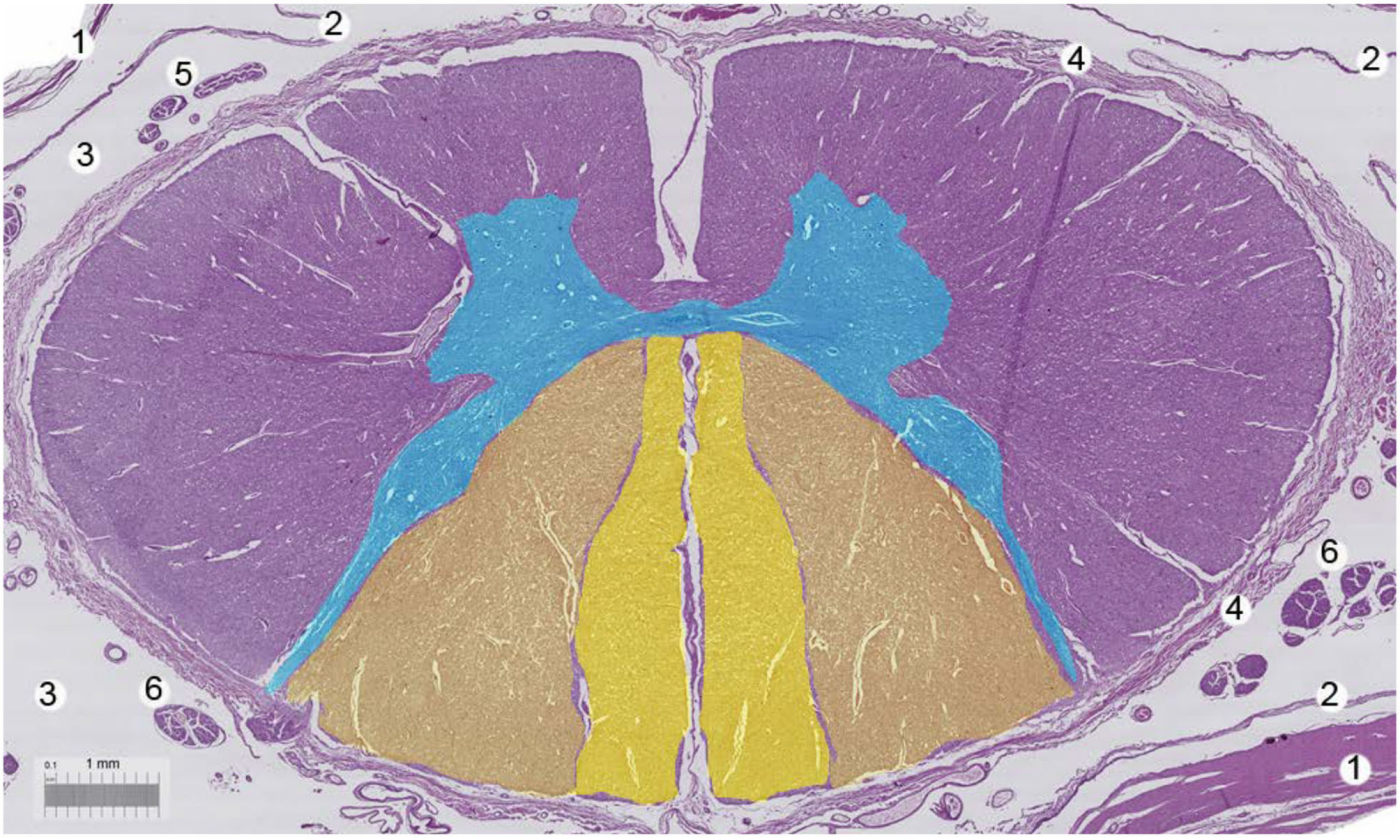
Human spinal cord. Bar, 1 mm. 1 = Dura mater, 2 = arachnoid layer, 3 = cerebrospinal fluid, 4 = pia mater, 5 = motor nerve roots, 6 = sensorial nerve roots. With permission of Dr. Miguel A. Reina.

**Figure 5:**
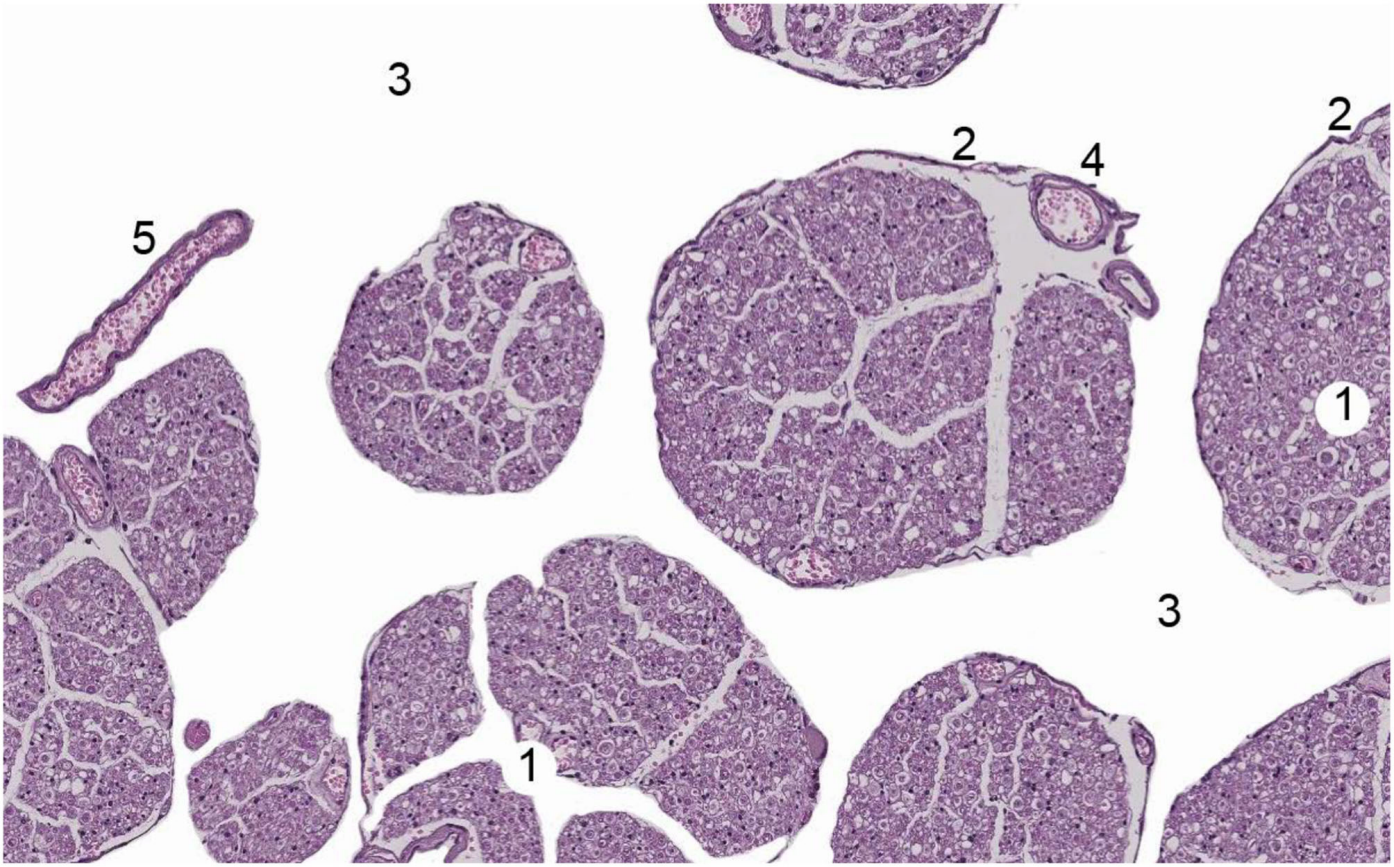
Human nerve root of Cauda Equina. 1 = nerve root, 2 = Pia mater, 3 = cerebrospinal fluid, 4 = vessel within nerve root, 5 = a free vessel within cerebrospinal fluid. With permission of Dr. Miguel A. Reina.

The spinal cord (Figure 4) and nerve roots (Figure 5) are surrounded by the pia mater, a poorly permeable layer formed by pial cells that restrict the passage of large molecules and particles from CSF into the spinal parenchyma. This cellular layer, presenting with a smooth and bright appearance, is made of flat overlapping pial cells (measuring, on average, 0.5–1 μm) (6) linked by desmosomes, resulting in the resistive barrier. Its thickness at the thoracic, lumbar, and conus medullaris level is 3 to 5 pial cells (10–15 μm). Two to 4 cells (3–4 μm) were encountered at the nerve root level. Behind the pia mater in spinal cord and nerve roots is the subpial compartment. It is enclosed between the pial cellular layer and a basal membrane, a limit membrane in contact with neuroglial cells, displaying a large content of collagen fibers, fibroblasts, a small number of macrophages, as well as blood vessels. At the level of the medullary conus, there are perforations or fenestrations over the entire surface of the cellular layer of the pia mater. These fenestrations have circular, ovoid, or elliptic shapes. While the dimensions of these fenestrations vary, most of them measure 12 to 15 μm in length and 4 to 8 μm in width. At the nerve root level, the pia mater also shows similar fenestrations but smaller in size (1–4 μm) (1, 10). Importantly, as noted above, while thin and fenestrated, the pia represents an important barrier for diffusion from the CSF into the parenchyma for large molecules and particles (4).

## Translational Investigation of Cerebrospinal Fluid Dynamics

### Cerebrospinal Fluid

The CSF contained in SSAS is Newtonian (e.g., its viscosity remains constant, no matter the amount of shear) and its viscosity ranges from 0.7.10^−6^ and 1.10^−6^m^2^/s, with molarity between 290 and 320 mOsm. Therefore, it is similar to water and 0.9% NaCl at 37°C. Its ionic content, low protein composition, and minimal cellular constituents have been discussed in detail in many texts (11).

### CSF Elaboration

The volume of the CSF has obvious relevance as a determinant of dilution of drugs in the SSAS. In man, about 500 ml of CSF is formed each day (12), mainly by the choroid plexuses of the cerebral ventricles, with significant contribution from the neuraxial parenchyma, reflecting the functional role of the glymphatic system (13–15). While the CNS has been considered to be devoid of lymphatic vessels, data in both rodents and humans demonstrate that lymphatic vessels are present and represent an extensive network along both the transverse and sagittal sinuses, as well as in sinuses at the skull base and, likely, the root sleeves (16–18).

### CSF Volumes

Classically, the entire CSF volume in man, including cranial and spinal levels, was defined as 150 ml, but Magnetic Resonance Imaging (MRI) has made a major contribution to allowing estimation of CSF volumes and movement from human axial images under physiological and pathological conditions (19–22), with significant variability between patients (12, 19, 20, 22–24). Of note, there is little or no correlation between patient weight and height and the entire CSF volume (20–22). In preclinical models, CSF elaboration rates and total CSF volumes have been reported, and these relative volumes have been used to estimate the translation of intrathecal dose/concentrations from the preclinical model to the humans (25).

## CSF Fluid Dynamics

Classically, formation of CSF in the choroid plexus and its absorption in the arachnoid villi were believed to create a “bulk flow” of CSF in the neuraxis (26). Such an assertion would then be considered reasonably to yield a uniform distribution of an intrathecal solute delivered to the lumbar spinal cord to distribute rostrally and to the brain. Examination of the local bolus or infusion of small lumbar volumes, however, clearly shows that this is not the case (27). We now appreciate that a fundamental property of the intrathecal space is that the fluid volume of the extracranial neuraxis, while not static, is a poorly stirred volume with the limited rostrocaudal flow.

### Oscillatory CSF Flow in the Spinal Cord

The characteristic property of neuraxial CSF is its oscillatory dynamics. This property is a key to understanding issues facing the delivery of neuraxial drugs and their behavior (28). This oscillatory flow, occurring in all animals, including humans, arises from pressure gradients in the CSF driven by two sources: cerebral perfusion and venous drainage.

- Cerebral perfusion leads to a volume of blood (~10 ml/heart beat) being delivered cyclically into the closed cranial cavity. This volume leads to a net caudal cyclical expulsion of a volume of intracranial CSF into the compliant extracranial (spinal) sac (28–30). Both the heart rate and the volume of the patient's CSF expulsed from the cranial cavity strongly influence the distribution of and intrathecally delivered solute. Doubling the heart rate (from 60 to 120 bpm) causes a 26.4% decrease in the maximum CSF concentration after injection (e.g., enhanced movement from the injection site). Doubling the stroke volume of CSF decreased the maximum concentration after injection by 38.1% (31). In addition to the heart rate, the CSF pressure waveform and flow are modulated by other physiological factors, such as the respiratory rate or increased abdominal pressure that impose separate pressure gradients on the compliant neuraxial space (see the next paragraph) (30).
- The extra-vertebral distribution of blood exiting the cord and entering the low pressure venous system of the thoracic and abdominal cavities may be reversed with increased intra-thoracic pressures (as with a Valsalva) that lead to compression of the venous return and back filling of blood into the large peri-spinal, valveless, venous sinuses (Batson's plexus) (32). This expansion of Batson's plexus in the lower thoraco-lumbo-sacral region has the effect of compressing the CSF volume in the extracranial CSF compartment, forcing CSF to move rostrally.

### Factors Governing Spinal Oscillatory CSF Flow

In the compliant volume provided by the spinal canal and the non-compliant spinal cord, the cranial pressure pulse can lead to oscillatory inflow and outflow at velocities up to 10 mm/s at the cervical cord level, with the velocity depending on the spinal canal diameter at any given spinal level (33). The amplitude of the rostrocaudal movement of these pulsations is about 9 mm per cycle in the cervical CSF and about 4 mm at the thoracic-lumbar junction, with minimal movement in the distal part of the lumbar sac. This gradient reflects several principles: (i) Flow is determined by conservation of mass flow. (ii) The spinal cord is only slightly compressible; however, the dural sac can expand practically to the degree limited by the rigid structure of the vertebral bodies and the contents of the epidural space (fat and vascular plexi). This compliance serves to accommodate the cyclical increases in spinal volume driven by intracranial blood volume. (iii) The absorption by the compliant dural sac of the energy/volume carried by the CSF pulse of the energy driving the fluid pulsation, thereby accounting for the rostroal to caudal reduction in the CSF pulsations. This property also suggests that increasing peri-spinal blood volume in the surrounding vascular plexi will not only generate its own pressure gradient in the spinal CSF; the decrease in the perispinal space decreases compliance, resulting in increased intrathecal pulse amplitude. Thus, the amplitude of the CSF pulsations is increased with elevation of intra-abdominal pressure (as with a Valsalva maneuver), leading to back filling of the vertebral venous plexi (34, 35).

This oscillatory movement, combined with the presence of the catheter and other geometric impediments in the SSAS (trabeculae, roots, and blood vessels) will lead to greater diffusion and convection that will enhance to a degree of the local dispersion of the injected solute. Computational models based on physical models incorporating local neuraxial geometry and impediments in association with the principle of CSF dynamics can serve to identify key variables for patient-to-patient variability in drug distribution in the spinal canal observed clinically (36).

### Spinal Cord Biomechanical Morphology

Computational analyses have indicated that the balance between local fluid acceleration and viscous forces produces a principal ordered flow, consisting of pure oscillatory rostrocaudal motion with axial velocities on the order of a few centimeters per second and amplitudes that, as noted above, decrease monotonically along the length of the spinal canal from cervical to sacral. The computational analysis also reveals a non-linear term associated with the convective acceleration that contributes to a constant flow that generates a local recirculating motion of the CSF along the channel with characteristic velocities two orders of magnitude smaller than the main one. This flow pattern is a key to the local neuraxial distribution of drugs in the SSAS (36, 37). Importantly, although poorly studied, eccentricities (lordosis and kyphosis) found in the human spine affect the magnitude and characteristics of slow bulk motion of CSF in the SSAS. Such deformities or other spinal injuries, leading to canal stenosis, can severely impact local solute redistribution (33, 38).

### Physical Complexity of the Intrathecal Space and Solute Movement

Nerve roots altered CSF dynamics in terms of the velocity field, constant flow, and vortex structures. Vortices were produced in the cervical spine around the roots during CSF flow reversal. The magnitude of constant CSF flow increased with nerve roots, particularly within the cervical spine. This increase was located axially upstream and downstream of the nerve roots due to the interface of adjacent vortices that formed around the nerve roots (39). Using Cynomolgus monkeys, the impact of SSAS catheter implantation on CSF flow dynamics has been studied. The Hagen–Poiseuille equation (describing the relationship between pressure, fluidic resistance, and flow rate) was used to investigate the impact of catheter implantation on flow reduction and hydraulic resistance. Results showed that catheter insertion had a significant impact on hydrodynamic parameters, altering lumbar catheter implantation to a lesser degree than cervical (40). Tangen et al., published potential guidelines considering drug-specific kinetics of tissue uptake of intrathecal drugs, which determine the speed of drug dispersion and influence tissue targeting (39, 41). It was shown that drugs with lower solubility advance 3 times more due to insufficient uptake in adipose tissue, or spinal tissue (39). Current computational capabilities are able to predict drug biodistribution mainly based on parameters like infusion settings, drug chemistry, subject-specific anatomy, and cerebrospinal fluid dynamics (31, 36, 38, 39, 41–43).

The development of computerized models based on fluid physics that can predict the biodistribution of agents administered intrathecally represents a potentially important approach to help physicians in choosing medications and schedule regimens and doses to improve treatment programs (44–48). These models provide the ability to perform representative *in silico* assays of subarachnoid injection and infusion protocols, including injection site location, injection rate, injection volume, and flush volume (34, 35). In addition, the models can assess the impact of physiological factors, including heart-induced increased CSF stroke volume and deep breathing (Valsalva) and posture (45). Understanding the anatomy and physiology of CSF dynamics is an essential element for managing the implementation of potential therapeutics for IDD in patients with neuraxial pathologies (e.g., pain, spasticity, and neuraxial infection/metastases.

## Translational Investigation of the Distribution of Intrathecally Delivered Drugs

On the surface, it might be anticipated that delivery of a therapeutic into the intrathecal space will readily lead to engagement of the appropriate intrathecal target. However, the behavior of an intrathecal injectate delivered into the intrathecal space with its restricted volume and its hydrodynamic properties reveals often unanticipated complexities.

### Local Intrathecal Drug Concentration Gradient

Following intrathecal delivery of a therapeutic, a rostral-caudal gradient from the catheter tip is typically observed. Such gradients have been observed in patients and in animals (46, 49). As an example, patients receiving IT infusions of morphine (39) sampled at varying distances from the infusion site showed CSF morphine concentrations, decreasing as a function of segmental distances from the catheter tip. These gradients are also evident from the dermatomal distribution of the density of block produced by spinal anesthetic (50).

The profile of this delivery gradient (height and spread) is regulated by several factors. (1) The height of this gradient is proportional to the concentration/dose delivered at the point source. In principle, the steepness of the rostrocaudal gradient is defined by the rate at which the solute is cleared from the intrathecal space. Such clearance into the adjacent meninges and parenchyma is governed by physicochemical factors, such as molecular weight, charge, and lipophilicity (40). Large, charged molecules are cleared slowly, while small lipophilic molecules are cleared rapidly and, hence, in principle, establish a relatively steep gradient around the drug delivery site. (2) The rostrocaudal spread of the gradient reflects the volume that has been delivered and the magnitude of the oscillatory flow, which diminishes from cervical to sacral and the velocity of the oscillations and is altered between cervical and lumbar by the relatively constricted thoracic space (51, 52).

Different three-dimensional computational models have been constructed for the investigation of CSF dynamics in the SSAS. These results manifest a porous media model that incorporates rostrocaudal (anisotropic) permeability variations within the SSAS. The integration of anatomical and velocimetric data with computational fluid dynamics principles allows reconstruction of precise fields of velocity and pressure in the investigated domain. Results are consitent with the physical biological model, showing the presence of global asymmetries in CSF flow, and that the net rostrocaudal flow through the spinal canal is insignificant within a cardiac cycle, despite the relatively large-amplitude CSF oscillation (53–55).

### Parenchymal Diffusion of a Molecule

Aside from a direct effect upon the meninges, an intrathecal agent much reaches its target, typically with the parenchyma, notably the dorsal horn for sensory and motor neurons for motor systems. As reviewed above, after IT delivery, the initial barrier to movement into the parenchyma includes the pia, which provides a barrier for high molecular weight molecules. In the parenchyma, the rate of movement is typically modeled by the diffusion, although the extracellular space, driven by the CSF-tissue concentration gradient. Such parenchymal movement is modeled as the rate at which a solute travels over a distance in free space and that rate required to traverse the distance in the tissue environment. This ratio is referred to as tortuosity. Variables that impact upon tortuosity are: (i) molecular size (larger globular solutes exhibiting slower diffusion than smaller, linear molecules), (ii) geometrical size (volume fraction reflecting the relative size of the extracellular space), and (iii) the chemical interactions between the molecule and surrounding cells or extracellular matrix. It is beyond the scope of this review to discuss the complexity of these issues here, and the reader is directed to learned reviews (56–58). However, the practical implications of the role of parenchymal diffusion to drug action can be seen in several examples reviewed elsewhere [see (59)]: (i) Time of the onset of analgesia in preclinical modelscovaries with spinal cord size (and depth to the dorsal lamina); (ii) Small lipid soluble drugs diffuse rapidly into tissue. It has, however, been long appreciated that, in this process, they are cleared rapidly into the parenchymal vasculature. So, as an example, while a lipophilic neuraxial analgesic (fentanyl) may show a rapid onset as compared to a hydrophilic agent (morphine), the ability of a lipophilic compound to reach deep parenchymal sites may be limited by being cleared as it diffuses and, accordingly, may require higher concentrations to sustain a concentration gradient to drive parenchymal diffusion in the face of its rapid clearance. (iii) With molecular weight, time of the analgesic onset also reflects changes in drug movement into the parenchyma with high molecular weight compounds, such as the calcium channel blocker (ziconotide), showing a delayed onset after delivery.

### Intrathecal DRG Access

As discussed above, the roots are invested in an arachnoid layer that terminates in a tight cuff proximal to the ganglion. This organization raises the question of how, following IT delivery, that even large molecules/particles (AAVs) can reach the cell-rich portion of the DRG (see below). Macroscopically, the dura and the arachnoid form a sleeve that continues to the proximal edge of the DRG. The epineurium, the outer most covering of peripheral nerves leading to the DRG, merges with the dura. The arachnoid is continuous with the perineurium and merging at the subarachnoid angle (60), which marks the end of the SSAS between the arachnoid and the pia. It appears likely that, at this point, the perineurium is open-ended to the SAS, and appears to be a likely location of communication between the CSF and DRG (61). More work is required to further define this linkage.

## Current and Future Analgesic Pharmacology

### Rationale for Spinal Targeting of Analgesic Therapeutics

The “pain experience” arising from exteroceptive (somatic/musculoskeletal/visceral) stimuli or events, which occur secondary to injury to the afferent-neuraxial pathways, reflects the complex integrative process that occurs at supraspinal levels (48). It is appreciated that supraspinal processes may enhance nociception through bulbospinal projections that drive enhanced dorsal horn excitability (62, 63). In all of these cases, a pivotal component of the pain experience strongly depends upon the content of the *spinofugal* message provided to the brain secondary to the spinal processing of the afferent traffic. This observation has been validated by the analgesic efficacy of spinally targeted pharmaceuticals (64, 65). We recognize that pain may have several orthogonal dimensions, classically referred to as the “sensory discriminative” and the “affective motivational” (66). It has been argued that changing the content of the spinofugal information content may impact upon the sensory-discriminative but leave unaltered the events underlying the affective motivation (suffering) component of the pain state. In this regard, we emphasize that altering dorsal horn afferent processing by spinal agents targeting a number of processing nodes clearly changes the content of the message being transmitted from the spinal dorsal horn to supraspinal areas believed to underlie affective processing (27, 67, 68), and so block not only the behavioral effects of tissue and nerve injury on not only evoked thresholds (e.g., allodynia and hyperalgesia) but also on activities that endow a highly rewarding phenotype to spinal drugs, which, otherwise, have no rewarding properties (69–71). Consistent with these preclinical observation are the reported improvements in wellbeing and emotional comportment in the human suffering from severe pain syndromes (64, 65). These joint observations point to the pivotal and defining role played, from a clinical perspective, in the efficacy and utility achieved by altering nociceptive processing at the levels of the dorsal horn.

### Intrathecal Drug Delivery of Analgesics and the Clinical Perspective

IDD allows for direct administration of spinally administered agents to their location of action within the CNS. IT drug delivery in principle has several advantages for the treatment of chronic pain as compared to peripheral delivery. IDD allows for the bypass of first-pass metabolism and a bypass of the blood-brain barrier. Due to the direct delivery of treatment drugs to the location of action, less drugs can be used, which can, in turn, result in less interaction with systemic receptors and overall less systemic adverse effects (45–48).

Many IT agents are utilized as the standard of care for the treatment of chronic pain *via* IDD, but only two IT drugs are approved for the treatment of chronic pain by the US Food and Drug Administration (FDA). Both morphine and ziconotide are approved as IT monotherapy for the treatment of chronic pain. The Polyanalgesic Consensus Conference (PACC) guidelines provide a framework to utilize IT therapy in a safe, efficacious, and evidence-based manner (72). The PACC guidelines are revised on a regular basis to address deficiencies and innovations in IDD. In the 2017 iteration of the PACC guidelines, disease-specific states, type of pain, and location of pain were taken into consideration (local vs. diffuse) (72). While there is FDA approval for the treatment of chronic pain using monotherapy with IT ziconotide or morphine and while there is evidence to show that monotherapy can be efficacious (73), it is routine for pain practitioners to report utilization of off-label medication and/or combination therapy (72). There are a number of determinants that have a significant impact on IDD. Important factors to consider are CSF flow dynamics, the pharmacokinetic profiles of individual intrathecal agents, rate of administration, drug volume, and placement site of the catheter tip (38, 44, 74, 75).

Important factors related to IT medications include drug dose, drug volume, rate of administration, and lipid solubility. Of the aforementioned drug factors, lipid solubility may be an important determinant as it relates to IDD at low flow rates. The depth of penetration by IT medications at the dorsal horn to exert their effect on target sites is on the order of 1–2 mm in humans (76). Hydrophilic drugs like morphine or large molecules (like ziconotide) are slowly cleared to the plasma and, hence, have a higher likelihood to maintain a driving diffusion to penetrate through the pia mater and diffuse to the superficial laminae of the spinal dorsal horn (substantia gelatinosa), although their rate of diffusion and, hence, time of the onset may be longer (40). Hence, as reviewed above, low molecular weight, hydrophilic, low lipophilicity IT drugs are more likely to have further rostrocaudal spread in the CSF and to penetrate into the cord as well. A hydrophobic drug like fentanyl will have a more limited spread within the CSF (e.g., a narrower rostrocaudal gradient as it undergoes rapid uptake clearance into the systemic circulation through the parenchyma and the meninges with absorption into the epidural fat (40).

### Intrathecal Analgesics

In the following sections, we briefly review the common currently approved intrathecal analgesics (72, 76–78).

#### Opioids

The *mechanism of action* intrathecal opioids is believed to exert their effects predominantly at lamina II (substantia gelatinosa) in the dorsal horn of the spinal cord (40, 79). It should be appreciated that, in addition, the intrathecal drug (opiate) may act directly to alter the excitability of the dorsal root ganglion neurons. This suggestion is predicated on two considerations. Even large molecules, such as antisense oligonucleotides and adenoassociated viruses, can impact the dorsal root ganglion after intrathecal delivery (see below). (2) *in vitro* studies have demonstrated that opiate can alter the excitability of the neuronal cell body (80, 81), and (3) increasing evidence suggests that the excitability of the DRG neurons can lead to an enhanced afferent traffic (82).

*Clinical utilization* opioids employed for intrathecal analgesia are almost exclusively mu opioid agonists that range from polar (morphine) to highly lipophilic (fentanyl). Aside from their mu-opioid efficacy, an important difference (as reviewed below) that some molecules (morphine) are strong activators of Mas receptor G protein coupled receptors (MRGs) and accordingly degranulate mast cells activate fibroblasts and may have a higher likelihood of yielding intrathecal meningeal masses) (83–86). Opioid medications are the most commonly utilized IT drug class whether used alone or in combination therapy for the treatment of chronic pain *via* IDD. While there are many opioid medications utilized in the IT space, morphine (Infumorph^®^) is the only FDA-approved IT medication for the treatment of chronic pain. The PACC guidelines make recommendation for all of the commonly utilized medications in relation to the level of evidence, recommendation grade, and the consensus level. The level of evidence and recommendation grade was based on the United States Preventative Services Task Force-created hierarchies (87). Consensus rankings (strong, moderate, weak) came from a determination by the authors of the PACC guidelines. In patients with active cancer-related pain, IT opioids are considered to have Level-1 evidence, a grade A recommendation, and a strong consensus level. For patients with non-cancer-related pain, IT opioids are considered to have Level-3 evidence, a grade B recommendation, and a strong consensus level (72). Additionally, the PACC guidelines consider morphine as a line 1A drug across all groups (cancer/non-cancer, diffuse/localized, nociceptive/neuropathic). The guideline also takes into consideration other opioids, including fentanyl, hydromorphone, and sufentanil, but these IT opioids are considered further down in the algorithm (72). While all of the opioids listed in the guidelines are utilized commonly in practice, the guidelines recommend FDA-approved IT medications as the first line.

While these opioids may have the same mechanism of action, some may be more advantageous when treating localized or diffuse pain. As noted, IT drugs that have lower lipophilicity and are more hydrophilic are able to penetrate further into the spinal cord and achieve wider spread in the CSF. Morphine and hydromorphone, due to their ability to spread and penetrate the cord, are recommended for diffuse pain. Conversely, drugs that are more lipophilic and hydrophobic like fentanyl may be more helpful in treating localized pain (74). Drugs like fentanyl diffuse quickly out of the CSF and may achieve plasma levels that mirror those observed after systemic delivery.

Traditional school of thought was that IDD was considered in patients on very-high-dose opioids or patients with cancer. More recent research has focused on IDD outcomes in patients who are either opioid naïve or on low doses of system opioids. Low-dose opioid IDD has been a topic of increasing interest and research as opposed to using high-dose IDD in opioid-tolerant patients. Several studies examined “microdosing” in IT therapy, which involves weaning a patient totally off or to very low-dose systemic opioid prior to trialing and implanting an IDD (88, 89). Once a patient has been weaned down or off opioids, the provider has the ability to utilize low-dose IT opioids. The PACC guidelines also review this concept of low opioid dosing for IDD, but further research in the form of randomized control trials is needed (78). While this concept of low-dose IDD is touched on in the PACC guidelines and described in the literature (88, 90), there is no current standardized or method of achieving low dosing recommended in the literature.

##### Adverse Effects

Potential effects related to opioid infusion include respiratory depression, peripheral edema, hormone changes, tolerance, opioid-induced hyperalgesia, constipation, urinary retention, pruritus, and intrathecal granuloma formation. Some of these side effects may be curbed with utilization of low-dose IDD in that a low-dose management strategy can lend to less overall side effects, decrease potential for opioid-induced hyperalgesia, and decrease the potential risk of granuloma formation at the catheter tip (see below for detailed discussion).

#### Ziconotide

##### Mechanism of Action

Ziconotide (Prialt^®^) selectively blocks presynaptic N-type calcium channels in the dorsal horn of the spinal cord (91). Blocking these calcium channels disrupts pain signal transmission by inhibiting the release of calcitonin gene-related peptide, glutamate, and substance P^92^.

##### Clinical Utilization

Ziconotide is the only non-opioid IT medication that is approved by the FDA for the treatment of chronic pain. Early clinical studies with intrathecal infusion ziconotide experienced significant side effects, which were attributed to over dosing based on a rapid dose escalation that failed to account of the extended duration of the spinal effect onset presumed to be secondary to the delayed movement of the molecule to the site of target engagement in the dorsal horn. The PACC guidelines consider IT ziconotide for the treatment of chronic pain (cancer/non-cancer, diffuse/localized, nociceptive/neuropathic) to have Level-I evidence, a Grade-A recommendation, and a strong consensus (2, 72, 73, 92). Deer et al. found that ziconotide as an initial drug for IDD resulted in improved pain control as compared to ziconotide being added as a rescue agent; however, the attrition rate—presumably due to ziconotide-related adverse events, was very high (93). The combination of morphine and ziconotide as initial IT agents has been recommended by both the PACC (72) and other studies (73, 94), although there is still room to thoroughly evaluate the benefits and associated risks of this combination.

While ziconotide has been found to be a safe drug that can provide pain relief, many times, its use is limited by potential side effects and narrow therapeutic window (95–99). Side effect to ziconotide may present early on during a trial or with up-titration. In many cases, ziconotide side effects may appear after the patient has been on a stable dose. Potential adverse effects can include psychiatric disturbance, dizziness, confusion, somnolence, myopathy, nystagmus, memory impairment, nausea, and abnormal gait (95–101). Dosing paradigms specifically designed to improve the safety and efficacy of ziconotide have been proposed: bolus (flex) overnight dosing and patient-controlled administration (102, 103).

#### Local Anesthetics

##### Mechanisms of Action

Local anesthetics block voltage-gated sodium channels in the neuronal cell membrane, which results in the blockade of action potential propagation (104). Unlike all of the other IT medications that target the dorsal horn of the spinal cord, local anesthetics are considered to exert their effects preferentially on the fila radicularia due to a large surface to the volume ratio of the rootlets as compared to that of the spinal cord (105).

##### Clinical Utillization

Bupivacaine is a highly lipid soluble amide local anesthetic and the only local anesthetic included in the PACC guidelines. In the most recent PACC guidelines, bupivacaine has been recommended as a 1B combination treatment with opioids in all treatment groups. The only drug recommendations higher than bupivacaine and opioid combination therapy are the two FDA-approved drugs (morphine and ziconotide) in all groups. Bupivacaine is only considered as a sole therapy in localized non-cancer pain (73). More commonly, bupivacaine has been utilized due to inadequate analgesia with opioid monotherapy, and it is considered to be the most common adjuvant utilized in conjunction with IT opioid (2, 5, 21, 34, 47, 78, 79, 83, 106, 107).

#### Alpha-2-Agonists

##### Mechanisms of Action

Clonidine is an alpha-2 adrenergic agonist that may be used intrathecally in the treatment of chronic pain. Activation of alpha-2 adrenoceptors has been shown to block noxious stimuli by pre- and post-synaptic mechanisms (108–110). Intrathecally, clonidine has been shown to inhibit the neuroimmune activation associated with neuropathic pain states (111). Clonidine more specifically can inhibit glial cells that add to enhanced pain states through the release of pro-inflammatory cytokines and inhibit the activation of NF-κB and p38 (111).

##### Clinical Utilization

The PACC guidelines recommend clonidine as a Line-2 treatment across all groups (cancer/non-cancer, localized/diffuse, nociceptive/neuropathic) (73). IT clonidine is not recommended to be used as a sole agent but, rather, in combination with an opioid, local anesthetic, or ziconotide. Clonidine is also recommended to be combined with two or more of the aforementioned drugs classes (72). IT clonidine has been shown to be efficacious in a dose-dependent manner in terms of pain control (111–113). At higher doses, there is better pain control, and cardiovascular effects typically stabilize (114). Clonidine has more cardiovascular side effects at lower dosing (114). Other negative effects that have been reported with IT clonidine include dizziness, dry mouth, bradycardia, confusion, hypotension, nausea, orthostasis, sedation, night terrors, depression, and insomnia (115). Sudden discontinuation of clonidine due to device failure or an empty pump can result in rebound hypertension, a serious adverse effect (116).

#### Baclofen

It is an agonist of the gamma aminobutyric acid (GABA)-B receptor, approved by the FDA, intrathecally, for spasticity (117). Animal studies indicate that baclofen has analgesic properties when injected into the spine (118, 119), and, in humans, it has shown efficacy, especially in patients with central pain associated with spinal cord injury (120–122), but showing lack of efficacy in complex regional pain syndrome (123). An animal study demonstrated that intrathecal baclofen reduced only the Phase-2 pain behavior after formalin injection, suggesting that the drug blocks noxious stimulus-induced spinal sensitization (124), but also modulating the effects of neuropathic pain in a nerve injury animal model (125). PACC recommendations (72) proposed with evidence Level II-2 as intrathecal medication for use to treat spasticity, also, with evidence Level II-3 recommend as an adjuvant to treat pain in Line 4.

##### Combination of Analgesic Therapies

The use of a combination of a local anesthetic and opioid medication has been widely described in the acute and chronic pain literature. Utilizing a combination of IT medications is a way to employ a multimodal approach to the treatment of chronic pain using IDD. It is generally accepted that a multimodal approach to pain control is superior to monotherapy. Combination therapy is commonly used because local anesthetics and opioid medications have been found to act synergistically when given IT in acute pain and animal models (126–131). The combination effects of local anesthetics and opioid medications in terms of pain control are mostly manifested as reduction of opioid dose escalation, which unfortunately plagues opioid IDD (132–134). When bupivacaine is combined with opioids at IDD initiation, it may help to limit the opioid dose escalation (135, 136). A number of studies have shown that 10 mg/day or more of IT bupivacaine can be helpful in the treatment of chronic non-cancer pain (135, 137–139). In one double-blinded randomized control trial by Mironer, there was no additional benefit when bupivacaine was added to opioids vs. the use of opioids alone (140). In the Mironer study, however, limited doses between 4 and 8 mg/day of bupivacaine were administered (140). Although long-term safety has been shown with bupivacaine infusions intrathecally in animal models, there are potential adverse effects, which include weakness, numbness, urinary retention, and hypotension (141, 142). One study with 82 patients being treated with IDD revealed significant decreases in systolic blood pressure and mean arterial pressure over 1 year (143). The decreased blood pressure associated with long-term thoracic IT infusion of bupivacaine is thought to be secondary to blockade of sympathetic efferents (143). The safety and efficacy of the low-dose intrathecal (IT) combination of ziconotide and morphine allows safe and rapid control of malignant pain refractory to oral opioids (94). This combination has also been recommended in the proposals of the PACC (72).

Intrathecal baclofen combinations have also been proposed for the treatment of chronic pain and spasticity. In line with the PACC proposals for its use as an adjuvant, reports have been made of its combined use (144), with clonidine (122), ziconotide (145), morphine (146), and bupivacaine (147, 148).

## Future Directions in Neuraxial Therapeutics

The richness of the dorsal horn pharmacology and the characterization of that pharmacology as being relevant to pain processing by the effects of the respective agonists and antagonists have been accomplished through neuraxial studies in a variety of well-defined preclinical models. These novel neuraxial therapeutic targets have been reviewed in detail elsewhere (59). Particularly notable has been the advances in the development of novel therapeutic delivery platforms to be delivered intrathecally to target a variety of neuraxial targets.

## Targeted Toxins

Targeted toxins have been developed, such as those developed using the botulinum toxin light chain, that reversibly alters transmitter release in the afferent pathway at the first and second order synapses (68), or toxins, such as SP-Saporin, that lead to cell death of the second order neurokinin 1 expressing projection neuron (149). Considerable work has indicated the efficacy of toxins targeted at specific receptors on the nociceptive afferent, such as for resiniferatoxin (150). Toxins arising from anthrax can be developed, which target specific sites on nociceptive afferents (151). Intrathecal delivery of these products has, respectively, displayed long-lasting, if not irreversible, effects, upon nociceptive processing preclinical models.

## Targeting the Genome

The targeting of the genome or message to alter the expression of channels and protein contributing to the processing of nociceptive information has been accomplished using antisense, viral vectors or lipid-based deliveries for CRISPR or zinc fingers or RNAi (152–156). Antisense oligonucleotides have virtue, given their ability to target messages leading to specific proteins. This platform has found significant implementation in neurodegenerative disorders, such as somatomotor atrophy (SMA), which are being successfully addressed by the use of FDA-approved intrathecally delivered antisense oligonucleotides (157, 158). These gene-targeting platforms requiring neuraxial delivery are exciting as they promise to produce long-lasting change in neuraxial processing at the level of the dorsal root ganglion or spinal dorsal horn that may be achieved by a single injection, perhaps foregoing the need for continued neuraxial access or delivery, importantly, the increasing utilization of the neuraxial route for novel targets and platforms.

## Advances in Neuraxial Delivery Platforms: Ports, Pumps, and Catheters

In managing chronic conditions (pain, spasticity, neurodegenerative pathologies), some drug-targeting platforms (such as transfection platforms or some antisense oligonucleotides) may require only a single injection to achieve an extended target engagement, while other approaches with molecules having a limited duration of action may employ indwelling catheters and IDDS. Although delivery into the brain and ventricles has been employed, the present discussion focuses on delivery into the spinal intrathecal space. The principal components required by such on-going delivery are the pump or port by which the drug is delivered and the delivery system itself, which is the implanted catheter. These systems are placed subcutaneously to avoid an exit wound for the catheter and enable continuous infusion. In the following section, we briefly consider the principal classes of systems and point to future directions.

## Pumps

There currently exist several pump systems. Here, we not specifically dwell on the specific pumps but the overall defining characteristics of the pumps that are currently available (159–161). Pumps are discussed further below.

### Construction

They are encased in a biocompatible shell (titanium), containing the drug reservoir, delivery mechanisms, and the power source, with the outer hull fitted with securing anchors.

### Drug Reservoirs

These reservoirs define the size of the implanted pump and may range from 20 to 60 ml.

### Access

Pumps typically have access ports, which allow multiple reservoir refills without leakage and, separately, access to the catheter downstream from the reservoir.

### Delivery System

Delivery of drug is driven by a pressurized portion of the drug reservoir, which provides a driving pressure, which is replenished when the pump reservoir is refilled with drug and/or by an electrically driven pump powered by internal batteries.

### Delivery Control

Output of the pump may be fixed or controllable. Controllable pumps may regulate the rate of pumping (e.g., as with a peristaltic rotor), producing a continuous flow or by sequential valves that allow filling of a small volume reservoir and then periodic expulsion of the minimum reservoir content with a rate of delivery controlled by recycling interval. Currently, the timing of delivery and the rate can be controlled by external telemetry.

### Rate of Delivery

Flow rates on the programmable pumps are, typically, in the range of 0–1,000 μL /h. Higher rates are typically used in a periodic delivery mode to achieve a pseudo periodic bolus.

## Catheters

Most of the implantable systems are provided with their respective approved catheter as a component of the implantable systems. There are several typical properties defining these implantable neuraxial catheter systems.

### Construction

Catheters should be inert, lacking irritants that may generate a local reaction. Important features include resistance to kinking and breaking. They have an external silicone covering and may be constructed from several layers, which include polyurethane and a radiopaque marker to facilitate identification of the catheter tip and, occasionally, along its trajectory.

### Size

**Figure 6:**
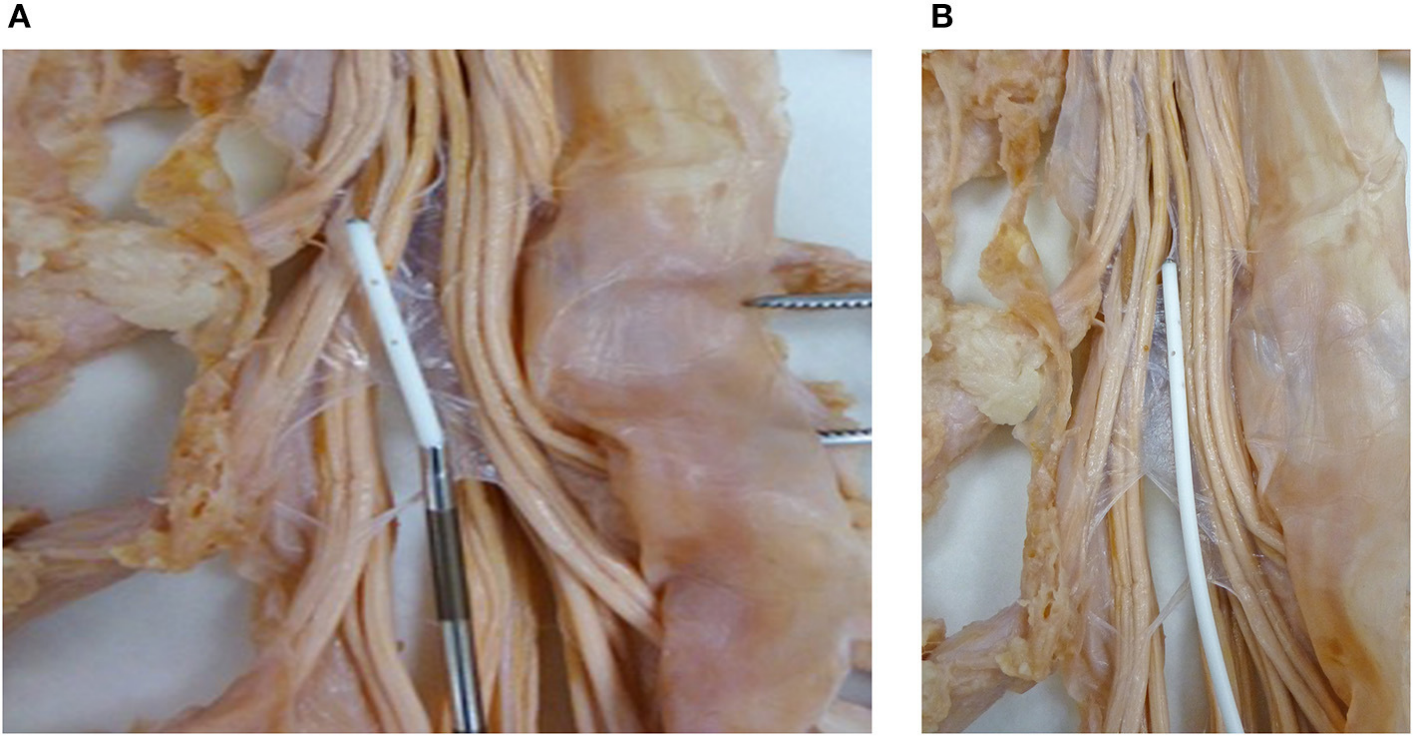
**(A)** Subarachnoid catheter. A catheter exiting the introducer needle in the vicinity of the roots of the cauda equina. **(B)** A catheter in the subarachnoid space to visualize the size relationship with respect to roots of the cauda equine.

Catheters approved for continuous use are on the order of 1.2–1.5 mm outside diameter, thus requiring a 15–16-G needle for their intrathecal placement (Figures 6A,B).

### Exit Port Configuration

Most implanted catheters display a closed tip and multiple side ports with the diameter of the side port similar to the internal catheter diameter (0.5–0.7 mm).

### Connectors

Catheters for chronic subcutaneous implant have specialized fitting that provides a secure leak-free connection at the pump outflow. Movable anchors are used to secure the catheter near its exit.

## Device-Related Distributional Properties of Neuraxial CSF and Injected Solutes

The previous commentary discusses the currently available delivery platforms. Let us now consider the issues related to the accomplishment of a satisfactory neuraxial drug delivery. As previously discussed in CSF dynamics section, since its initial use in humans for anesthesia (162) and then with infusion for pain (163) and, later, for spasticity (117), our understanding of the principles governing the delivery of drugs to the spinal cord has grown. In summary of an extensive literature summary overview:

The spinal cerebrospinal fluid space is composed of a closed cranial and extracranial (spinal) fluid space, displaying significant compliance (164, 165).

- Cerebral spinal fluid (CSF) is formed in the cerebro-ventricles and by movement from along the parenchyma along the spinal into the intrathecal space (15).
- The movement of spinal CSF is driven by (1) the periodic pressure gradient established by expression of CSF from the brain as a result of filling with blood during the systolic phase of the cardiac cycle, resulting in a limited cranio-caudal/caudal- cranial CSF movement (as permitted by the compliance of the spinal dural sac located within the boney spinal canal and (2) by periodic increases in intrathoracic pressure with respiration, which increases compressive blood volumes in the peri-spinal venous sinuses (Batson's plexus) (41, 166).
- These local oscillatory movements are greatest in the cervical spinal region and, progressively, more limited as one proceeds caudally (167, 168).
- The intrathecal space is a complex environment with significant physical impediments to free CSF movement flow. These impediments are the numerous septae, connecting the dura arachnoid to the pial surface, which may partition to varying degrees the intrathecal volume into adjacent spaces, the trabeculae that connect the dura arachnoid to the surface of the cord, traversing blood vessels and roots (41), and the symmetric location of the spinal cord in the spinal canal, resulting in a complex streaming pattern (169).
- Given these properties, two characteristic parameters of injectate movement are noted. First, at the drug delivery site, a low rate or volume results in a stagnant pool of injectate proximal to the catheter tip results in the exposure of local tissues to the infusate concentrations being delivered. Second, the rostrocaudal distribution of a dye marker or drugs delivered into the lumbar space is thus limited with the local injectate, displaying a declining rostral and caudal gradient around the intrathecal injection site with the peak concentration at the injection site and the tails of the rostrocaudal distribution proportional to the infusate drug concentration (74, 75). Over time, if the drug is poorly cleared from the CSF space (as with large molecules or particles), the local cyclic pressure gradients will lead to gradual dilution of the infused material and a rostro caudal spread.
- Increased rostrocaudal movement and increased local mixing of an injectate may be accomplished with higher infusate volumes or higher rates of infusion (47). However, the limited volume of the CSF space restricts the volume that may be delivered by bolus or infusion. For the implanted human pump with a limited reservoir capacity (20 ml), continuous spinal infusion programmed rates tend to be low, and total daily volumes typically do not exceed 20 μL/h to increase the interval between percutaneous refilling of the pump.

## Consequence of Restricted CSF Redistribution

As noted, spinal drug redistribution represents two dimensions: (i) movement rostrally and caudally from the site of delivery and (ii) the local dilution of the injectate that exits from the catheter tip. These two components contribute to two issues of relevance to the effects of intrathecal drugs.

### Spinal Drug Activity: Role of Rostrocaudal Redistribution

In many cases, the target to be engaged by the intrathecal drug may extend over several segments. Consider two scenarios: First, therapeutics to target neurodegenerative changes or central seeding cancers may require intrathecal drug distributions that extend from sacral to cervical cord; second, spinal processing of nociceptive input and/or changes in spinal function leading to spasticity is not limited to a single spinal segment. Thus, afferent traffic from a single nerve root may send collaterals to spinal levels up to 5–10-segment distance (170). Accordingly, spinal drugs (other than a local anesthetics that act upon local nerve roots), such as morphine must reach the terminals of the afferent or distal dorsal root ganglia (as with an adenovirus transfection) delivered the lower lumbar may necessitate reaching thoracic or even cervical spinal segments. Absent a prominent CSF movement after delivery from a point source delivery leads to a restricted movement of the infused drug and a rostrocaudal gradient that fails to engage distal targets relevant to the spinal processing required to regulate pain or spasticity state. We argue that these characteristics result in the need to employ higher concentrations (to increase the drug exposure at the rostrocaudal tails of the intrathecal distribution curve and to increase drug-delivery volumes).

To enhance rostrocaudal spread, larger volumes and/or rates of infusion are typically considered appropriate. While intuitively reasonable, using the anesthetic level to assess drug distribution after intrathecal delivery of local anesthetics reveals that increases in spinal levels are only modestly increased with increased intrathecal volumes (50). For bolus delivery, the rate of infusion does not appear to reliably affect the degree of rostral distribution as measured by block height (171–173). This limitation on the effect of volume and bolus delivery upon rostrocaudal movement reflects upon two properties: (i) injectable volume is restricted. Studies employing intrathecal delivery of local anesthetics are typically limited to 10–20 ml, and the estimated spinal CSF volume is 90–120 (24, 173). So, the ability to force rostral drug exposure with increasing volumes is usually limited to, perhaps, at most, 20% of the spinal volume. As regards to the rate of delivery, the compliance of the dural saccauses the energy imparted by a bolus infusion to be minimized. Furthermore, the rostral movement of the fluid stream is hindered by the complex impediments present in the intrathecal space. Furthermore, low-infusion, low-volume regimens accentuate the limited rostrocaudal movement of the drug. One consequence of this lack of active rostrocaudal redistribution is to increase the required infusate concentration (given the inability to increase volume to increase the length of the rostrocaudal gradient).

### Spinal Drug Activity: Role of Local Redistribution

Low volume/low rate infusion in the absence of robust local CSF movement results in local tissues to be exposed to highly concentrated injectate. Several examples emphasize that such local exposure leads to an enhanced likelihood of local toxicity (25). Two examples are well-understood: First, the incidence of root and nerve injury yielding a radiculopathy may occur after intrathecal local anesthetics. The incidence of this pathology is enhanced with high concentrations formulated as to resist redistribution (hyperbaric solutions). The lack of redistribution is accentuated by the use of “microbore” catheters, which restrict the rate at which the drug can be infused (174); second, meningeally derived space-occupying masses composed of meningeal fibroblast and a collagen matrix may occur in guinea pigs, dogs, sheep, and humans after the infusion of high (FDA-approved) concentrations of several opioids (83–85). This phenomenon has been shown to evolve in a *concentration*-dependent fashion (83). It has been shown that the pathology results from the poor redistribution of a slowly delivered infusate, which minimizes the redistribution at the catheter tip. Increasing the rate of delivery using or the use of low concentrations, strategies that increase redistribution around the catheter infusion site, serves to diminish the incidence of the intrathecal mass. In recent work, it has been shown that delivering a fixed volume of concentrated morphine as a continuous infusion (20 μL/h) leads to spinal masses. In contrast, delivering the same total dose/volume in multiple-divided fast boluses resulting in greater local movement reduces the incidence of such masses (175).

## Future Development in Neuraxial Drug Devices and Delivery Strategies

There are two questions: (1) how to increase local redistribution around the catheter tip and (2) how to improve the rostrocaudal distribution of an injectate where permissible volumes are limited. Future direction in system development suggests two strategies to address these issues: a drug delivery profile and catheter configuration.

### Increased Local Redistribution Around the Catheter Tip

**Figure 7:**
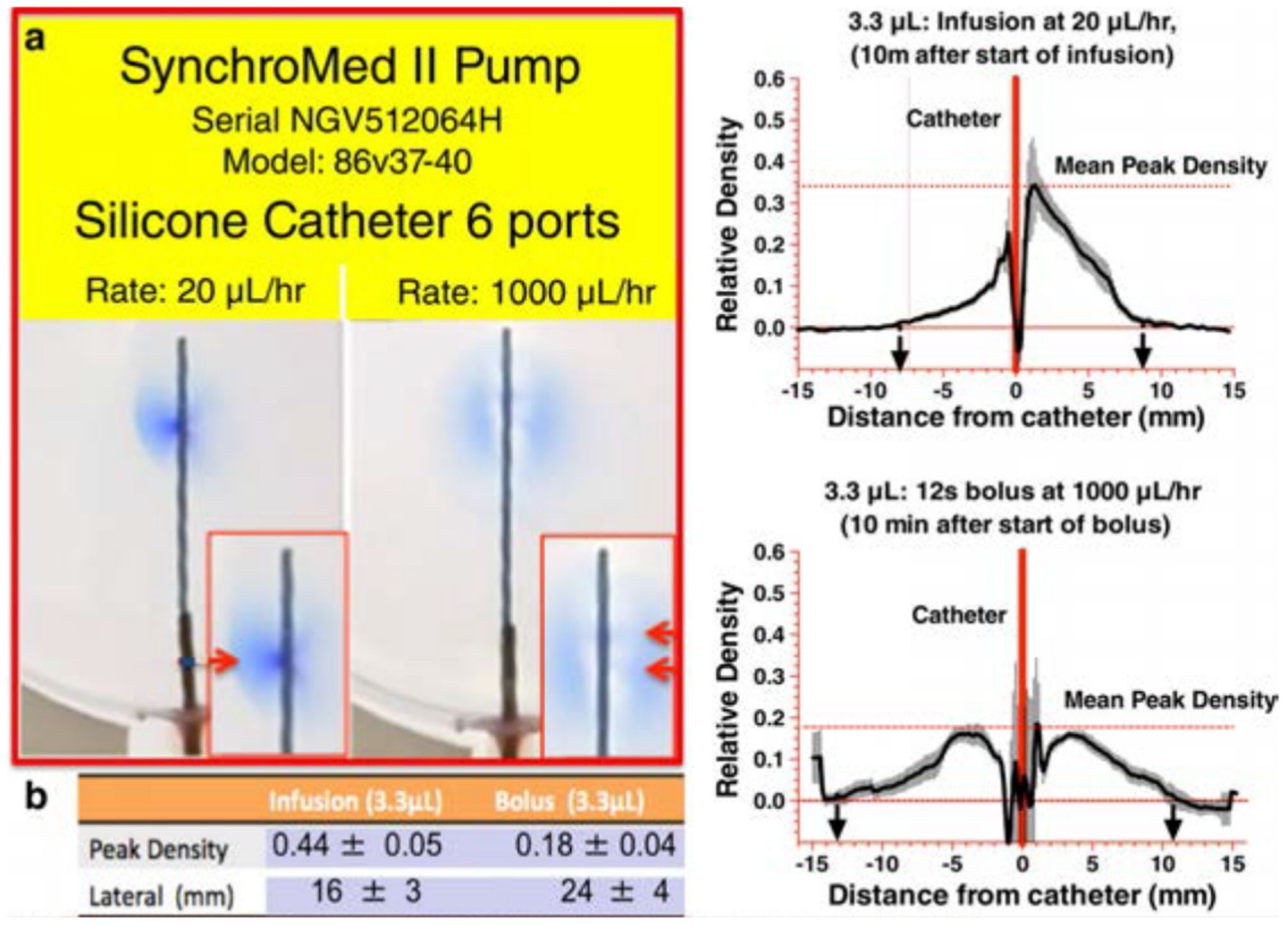
[**(A,B)** Left] A photograph of the 2D diffusion chamber, showing movement of blue dye from the catheter (Model 8709SC) connected to a SynchroMed II pump. The most proximal (to the pump) pair of ports is located at 180° from each other, and the catheter is arranged so that the axis of these first two ports is parallel with bottom of the chamber. An image taken at 10 min after the start of a continuous infusion of 2% methylene blue dye at 20 μL/h, delivering 3.3 μL. (Right): 10 min following the bolus delivery of 3.3 μL at 1,000 μL/h. b. Mean ± SEM (*N* = 5 replications) densitometry measurements (arbitrary units) across a line perpendicular to the catheter at the point of dye exit from the first port proximal to the pump as shown in the left and bolus as shown in the right [reprinted by permission from Hildebrand et al. (175), Wiley].

i) Drug infusion profiles: As noted, delivery of low volumes at low rates of infusion results in a poor local redistribution, promoting potential toxicity. Enhanced redistribution may be achieved by increasing the exit velocity from the catheter. Exit velocity is a function of two parameters: (i) the volume that is expelled per unit time and (ii) the size of the orifice through which the volume is expelled. Thus, for a given volume/unit time and modeling the orifice size as resistance to flow, exit velocity ∞ R_exit_; the greater the exit velocity, the greater is the directional spread. The demonstration of the enhanced delivery resulting from a microbolus vs. a continuous infusion is presented in Figure 7. This *figure* shows typical densitometry profiles for a dye delivered into a planar diffusion cell for a single bolus of 2.6 μL/h after 8 min vs. continuous delivery (20 μL/h for 8 min = 2.6 μL). As both pumps have delivered the same volume (~2.6 μL), the difference in tip-dye density shows that the single micro-bolus leads to a greater local redistribution at the catheter tip than the distribution produced by the continuous delivery profile. This is consistent with higher exit velocities at the catheter tip after bolus delivery. Based on our modeling of exit velocity, 1/2 height diffusion distances and local catheter tip dye concentrations, we hypothesize that increased distribution, reduced density, and increased mixing will occur with large micro-boluses (20 μL/bolus) > cumulative microboluses (1–5 μL/bolus) > continuous infusion.

ii) Catheter design: *Orifice size*: As exit velocity ∞ R_exit_, then it is apparent that, for a given flow gradient, the higher the resistance to exit, the greater will be the exit velocity. As noted above, the typical human catheter employs large exit orifices and, with a low-rate infusion, the majority of the infused solute exits from the first orifice and even with the bolus delivery, the exit, although enhanced, is still limited to the first pair of ports. In contrast with the same delivery through smaller ports, the exit velocity is markedly enhanced, as is the distribution. The limitation of small orifice is that the ability to withdraw may be restricted.

### Improve Rostrocaudal Distribution of an Injectate

**Figure 8:**
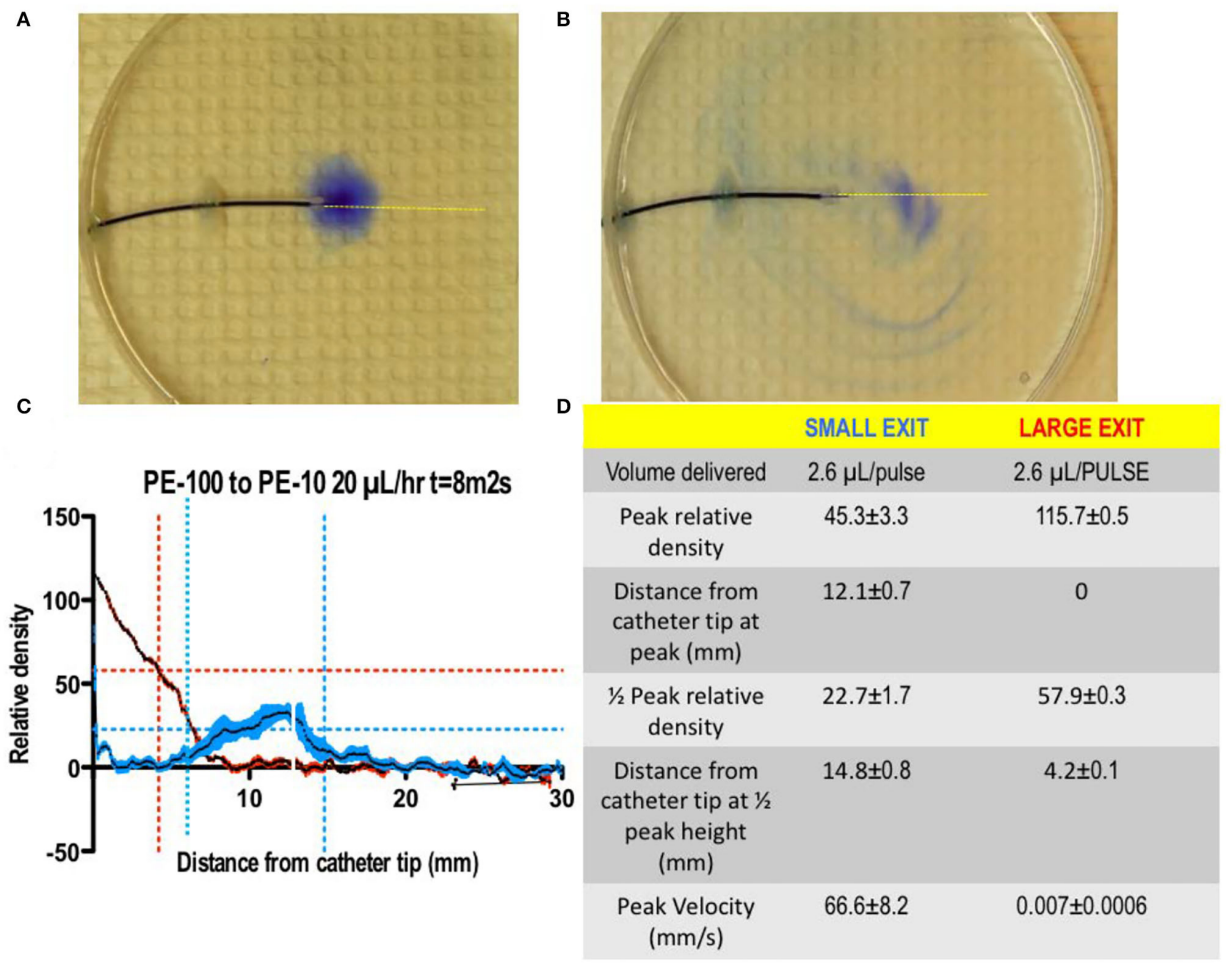
**(A,B)** A photograph of the 2D diffusion chamber, assessing movement of a small volume of blue dye (2.6 μL) delivered as a bolus through A: a small orifice (0.011” dia) vs. a large (0.34” dia) orifice. As indicated in **(C,D)**, the delivery through a small orifice resulted a high exit velocity with a reduced peak concentration and the shift of the peak concentration away from the catheter lumen as compared to the bolus delivery of the same volume of dye through a large lumen (T.L. Yaksh).

**Figure 9:**
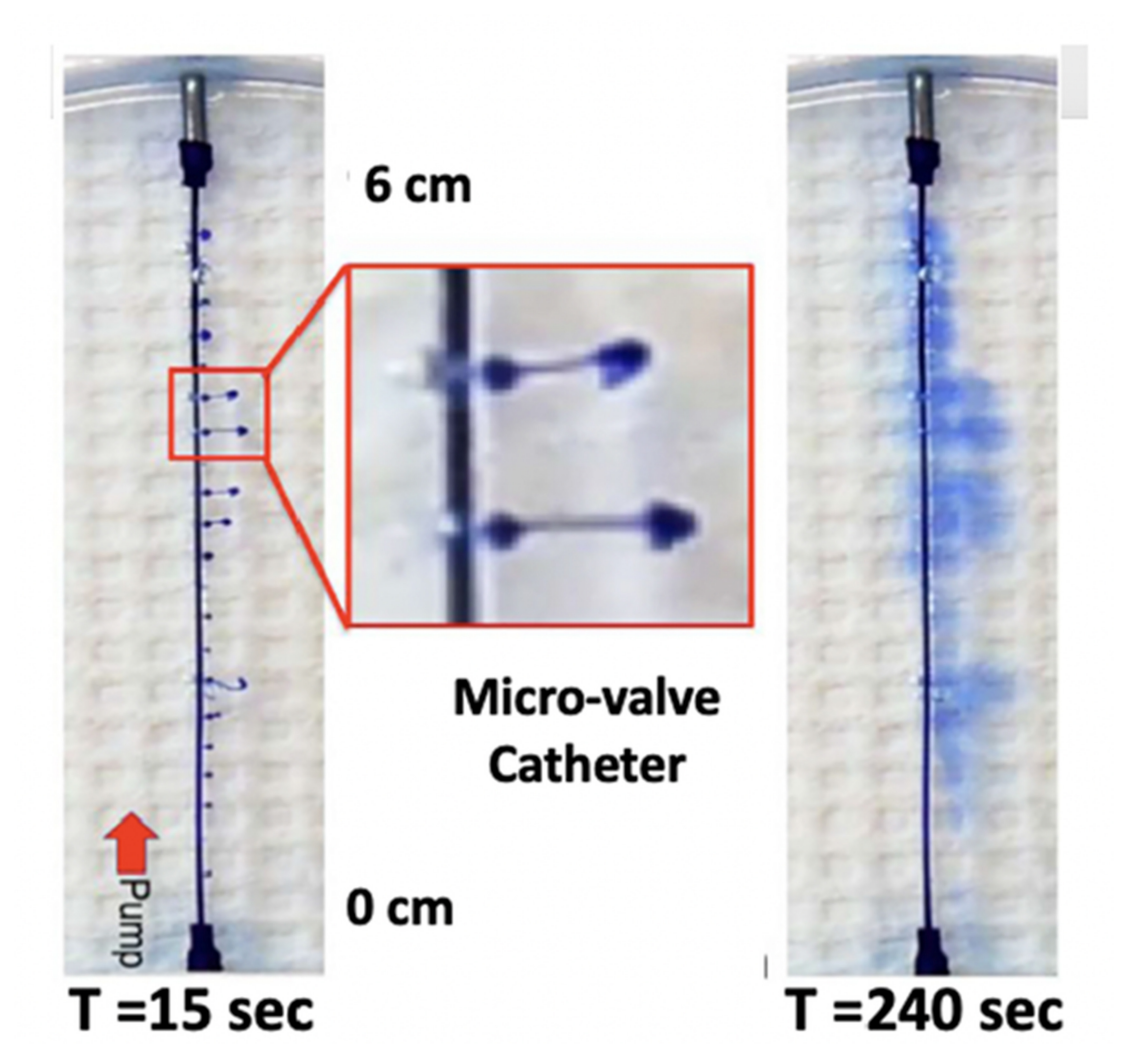
Two-dimensional diffusion chamber with a catheter having 20 microports. Images taken at 15 s (top, left) and 240 s (bottom, left), following bolus delivery of 2.6 μl as a bolus at time 0. The image on the right of the T = 15 s image shows an enlargement of the high velocity stream exiting the catheter at two of the exit valves, with the characteristic mushroom head where dye laden solute encounters the local dye-free fluid phase. Note the even distribution of dye from proximal (pump) to distal over the 6-cm catheter distance (T. L. Yaksh).

i) *Multiple orifices:* If the total injectate could be delivered over a length of catheter, the total volume would be divided over the length of tubing (instead of a single point source). This would achieve the aim of distributing a given volume of injectate over a larger rostrocaudal length of cord and resulting diluting smaller volume of injectate in larger local volumes of CSF (thereby achieving the aim of reducing local drug concentration). To achieve that distribution, as demonstrated in Figure 8, the pressure head at each port would have to be the same along the length of the catheter. Large ports would result in the total loss of resistance at that proximal site, and all of the injectate would exit there. By using very small ports, the cumulative cross-sectional area being small compared to the catheter diameter, all micro ports would see the same pressure head, and the amount of injectate delivered through each port would be the same. This is emphasized in Figure 9, showing an even distribution of a very small volume (2.6 μL) over a 6-cm length of delivery. The principle, as described, applies to catheters of longer length and permits the even distribution of a small volume over an extended interval and reduces the need to employ large volumes of a high concentration to reach distal sites from the site of delivery.

ii) *Multiple lumens:* The catheter employed to day for neuraxial drug delivery typically employs a single lumen. Given available mono reservoir drug-delivery devices, the present practice consists of pre-mixing multiple drugs for simultaneous spinal delivery. However, such concurrent delivery faces issues of different pharmacokinetics and side effect profiles of the components of the admixtures and presents concerns.

**Figure 10:**
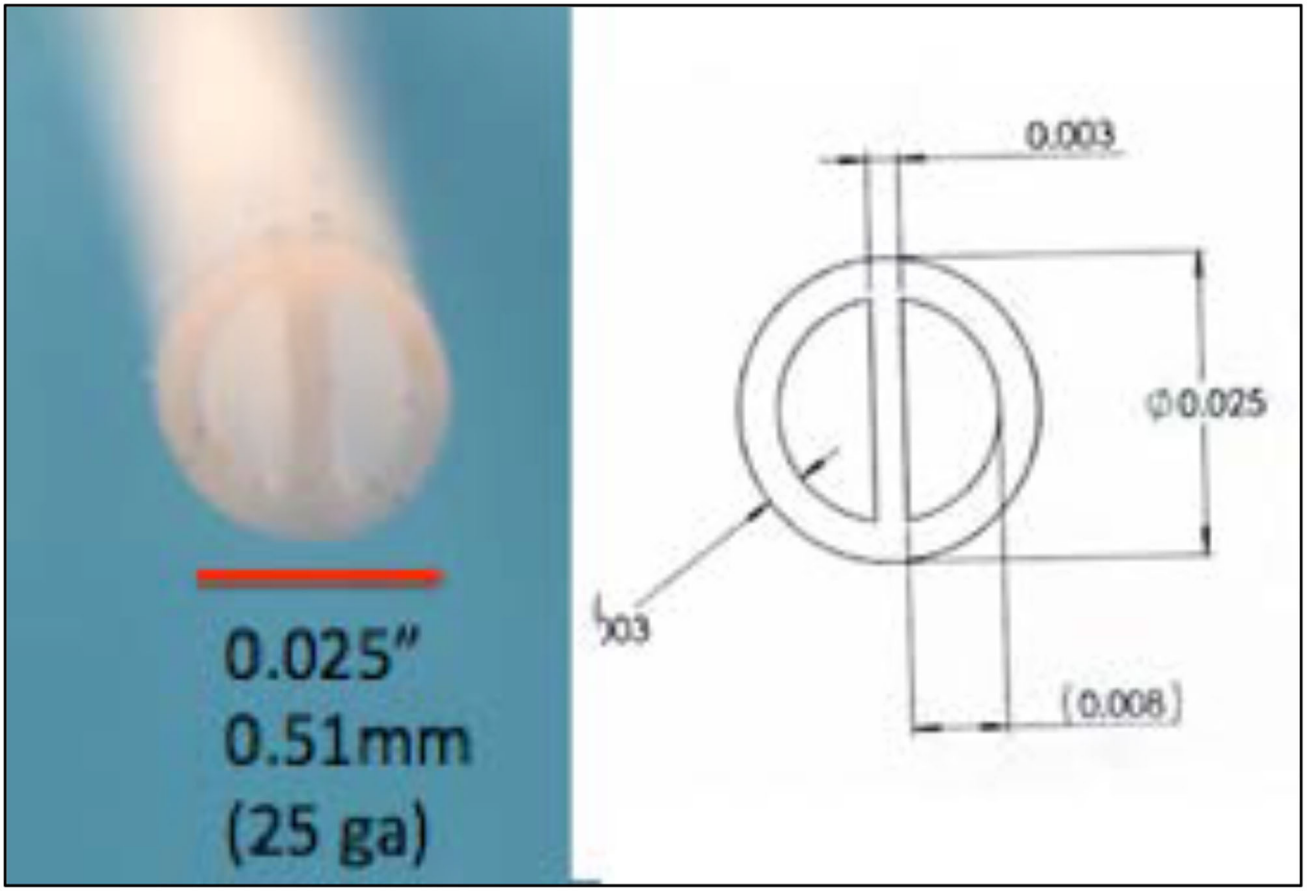
A double lumen catheter (Polyurethane) for intrathecal placement.

Alternatively, it is appreciated that many pathology states may be time variant, e.g., an ongoing pain state and brief intervals of “incident” pain. Here, the aim is to use a long-lasting ongoing infusion as a background medication with an alternate fast onset and brief acting agents for control of the incident conditions. Such combinations might include morphine and fentanyl. However, mixing the two products obviates the slow and fast time courses required from controlling ongoing and incident pain. The implementation of such a dual lumen catheter, however, requires alternate catheter designs, notably the use of a dual lumen catheter. Figure 10 shows a 0.025” double lumen catheter suitable for preclinical placement.

As a summary of this section, the current neuraxial delivery systems permit addressing the need to deliver therapeutics over long periods of time. However, it is clear that the standard components, although increasingly sophisticated, have changed little in principle over the past 50 years. Given our understanding of neuraxial injectate distribution, it is clear that further advances may benefit from a rational consideration of the role played by catheter orifice configuration and the role of exit velocity in enhancing local distribution and enabling the even distribution of infusate over extended intervals of the spinal axis.

## Delivery Programming and Outcome

As reviewed in the previous section, the development of systems, which can deliver programmed infusion as boluses and/or continuous delivery at different rates, suggest the potential impact that programming can have on neuraxial drug distribution. An important question is whether these programming choices, indeed, lead to changes in a clinical outcome. The influence of the injection rate on drug distribution is well-known by anesthesiologists performing spinal anesthesia. To allow a wider sensitive block, local anesthetic has to be injected faster, with injection withdrawal of small volumes (barbotage). Compared to the classical injection of 2–3 ml over 30 s during a spinal anesthesia, the kinetic energy contained in an injectate during a typical 20 μl/h continuous flow rate is about 85,000,000 times lower for chronic intrathecal administration. Therefore, increasing the amount of kinetic energy, with a faster injection rate from the catheter, will improve local drug distribution in the cerebrospinal fluid (CSF). In preclinical large animal studies, Bernards and colleagues compared three different rates of administration of Bupivacaine, Baclofen, and Methylene blue: continuous, 20 μl/h; continuous, 1,000 μl/h; and bolus, 1,000 μl over 5 min every hour (46). After 8 h of administration, drug infusion at 20 μl/h was very limited. The faster infusion rates in the 1,000 μl/h group and, mainly, in the bolus group did impart moderate but observable, forward motion to the injectate, and differences among the groups in drug distribution might be, in part, the result of differences in kinetic energy associated with the different infusion rates. However, a 1,000 μl/h injection rate is not conceivable in clinical practice since it would lead to very short refill intervals, <2 days for a pump of 40 ml.

In humans, several studies failed to show any improvement in pain relief by only increasing the continuous flow rate without modifying the daily dose. In a study by Perruchoud et al., twenty patients on stable intrathecal treatment were included in a double-blind three-period crossover study where the same daily dose was administered at single, double, and quadruple flow rates in a randomized sequence (176). Visual analog scale scores remained unchanged with all flow rates. The quadruple flow rate even worsened the quality of life of the patients compared to the single flow rate. Increasing the flow rate without changing the daily dose may have resulted in local dilution of the drug at the site of action without increasing the distribution along the spinal cord. In a similar double-blind design study, van der Plas and colleagues evaluated the effect of varying the injection rate at a fixed daily dose on the efficacy and safety of intrathecal baclofen in patients with CRPS-related dystonia (123). The patients were randomized to either slower infusion rate delivery or four-times faster infusion rate delivery for 2 weeks and were then crossed over after a 1-week washout period. The authors found no significant differences between the injection rate regimens in terms of dystonia or pain and secondary outcomes, except for the frequency of adverse events, which was significantly higher during a faster infusion rate.

The first device allowing the patient to self-administer bolus in advance of expected increases in pain or in response to sudden breakthrough attacks was launched in 2004. Since then, several studies have evaluated the effectiveness of a patient's control of the intrathecal analgesia (PCIA) or a personal therapy manager (PTM). Buchser and colleagues described the improved pain control and clear thermoanalgesia obtained by the addition of small, presumably negligible, bolus doses in two patients with cancer who failed to get relieved by a seemingly significant daily dose of bupivacaine administered as a continuous intrathecal infusion (177). A multi-center study, which included 168 patients with chronic cancer and non-cancer pain treated by intrathecal drug application by means of programmable pumps and the above-mentioned PTMs, showed very good acceptance of the device after a 12-month period (178). The patients were able to manage unpredictable pain fluctuations very actively, easily, and safely, and also learned to use the device to control expected pain fluctuations by prophylactic recall of boluses. Overall satisfaction and acceptance of the PTM by patients were >80%. In a retrospective study in refractory cancer pain population, Brogan and colleagues reported that 50% of the patients had discontinued all non-intrathecal opioids at follow-up, and 46% of the patients on breakthrough medications no longer required their use after initiation of intrathecal administration with PCIA (179). The same author conducted a prospective study, including 58 patients with refractory cancer pain (180). PCIA was associated with improved pain control (superior analgesia and a 3-fold faster onset of action), improved cancer-related symptoms, and high satisfaction compared with conventional breakthrough pain regimens. In another retrospective study comparing the need for oral analgesic opioids to manage unpredictable breakthrough pain in patients treated with intrathecal pump for non-malignant pain, 11% of the patients using the PTM continued to require oral opiates to manage breakthrough pain compared to 57% without the bolus option (181).

As discussed above, periodic bolusing administration thus might be an interesting alternative in terms of higher kinetic energy input, better drug distribution, and improved therapeutic effects. Superiority of bolus compared to continuous administration in terms of drug distribution has been demonstrated in other anatomical compartments, such as epidural (182), or paravertebral spaces (183). In a separate small pilot study, 10 patients with chronic pain were randomized to two periods of continuous administration of intrathecal opioid or bolus regime (40% of daily dose split into four equal boli applied every 6 h, with the remaining 60% as background continuous infusion), in a crossover fashion (184). Overall, bolus periods were associated with a small but significant reduction of numeric pain rating scores (mean −0.56; *p* < 0.0001). Preferential bolus administration, with only a minimal continuous flow rate, has been evaluated in a small case series (*n* = 4) published by Heetla (185). Spastic patients who developed tolerance with continuous intrathecal administration of baclofen were switched to bolus administration of the same daily dose divided in 6 boluses. Bolus administration resulted in the stabilization or decreasing of the daily intrathecal baclofen dose in all four patients during the follow-up period of 12 months, whereas the spasticity scores remained stable or even improved during the same period. Among the hypotheses put forward to explain these results is a better distribution of the drug in the CSF as well as the resensitization of GABA-B receptors made possible by the intermittent fluctuations of CSF concentration of baclofen between boluses, acting as “drug mini-holidays.” This observation was, however, not replicated by a prospective randomized cross-over study, evaluating the switch from continuous to intermittent bolus administration of the same daily dose in patients with chronic pain (186). The mean patient global impression of change (PGIC) and proportion of positive responders were not substantially different after intermittent bolus vs. continuous administration.

The influence of different administration patterns had also been evaluated for intrathecal trialing prior to the placement of an implanted device. In a prospective/randomized study, Hamza compared intermittent boluses to continuous infusion prior to the pump implantation for the treatment of severe intractable chronic non-malignant pain (187). The results failed to show any clinical significant difference between the two methods in terms of predicting trial success or long-term outcomes.

The incidence of granuloma has been reported to be significantly less frequent with bolus administration compared to a continuous flow rate, meaning that bolusing increases local pericatheter dilution (but not rostral distribution of drugs) (175).

As reviewed in the preceding sections, the two commercially available programmable pumps employ a peristaltic driver or a valve-gated device (160). With the peristaltic pump, the roller assembly, which, with its rotation, provides a continuous outflow of the medication. The valve-gated pump incorporates a positive pressure design, consisting of two microvalves, a dosing chamber and a flow-activated valve. In this system, drug delivery is achieved *via* positive pressure, propagating the drug through the open inlet and into the dosing chamber. The inlet valve then closes, and the outlet valve subsequently opens, allowing for drug delivery in a small and fast bolus (typically on the order of 2.5 μL/pulse). The effects of these two delivery profiles on efficacy and distribution remain to be determined (see the previous section).

## Selection and Indications for Intrathecal Therapy in Chronic Pain

Selection of appropriate patients for IDD is critical to successful outcomes. Many patients may be referred for or desire IDD for their refractory pain, but only a select few may be appropriate for treatment with IDD. Patients are often considered for IDD when they have objective pathology to treat, have failed other conservative measures to manage their pain, or are unable to tolerate other forms of opioids. Patients typically undergo psychological testing with a mental health provider and must exhibit a favorable psychological profile (188, 189). While the aforementioned factors are important, newer literature has revealed that other factors, including patient demographics, medical history, use of opioids, and pain characteristics, should be taken into account.

When it comes to patient demographics and medical history, a number of factors, including age, gender, certain comorbid conditions, and concurrent medications, should be considered. It has been shown that oral opioid doses escalate, and tolerance develops quicker in younger subjects vs. older (190, 191). Hayek et al. found that dose escalation with IDD occurred much faster in patients younger than 50 years old. Older patients in the study by Hayek et al. had less dose escalation with IDD and showed a significant decrease in concurrent oral opioid requirements (133).

Gender has shown mixed results in terms of being a determinant for IDD outcomes. One retrospective study of 86 patients revealed that escalation of IT opioids occurred at a lower rate in women vs. men (132). Women more specifically had lower total daily IT dosing at 18 and 24 months (130). Other studies have not reproduced the same gender-related differences in IT opioid dose escalation (89, 133, 192).

While, often, the focus is on treating pain, when considering an implantable device, attention must be directed to the patients' co-morbid conditions and how they may impact IDD. Patient factors that may impact care include glycemic control, smoking, immunosuppressants, chemotherapy agents, anticoagulation, and concurrent infection or colonization. Poor glycemic control has been associated with increased risk of surgical site infections (SSI). In the spine literature, a high HgbA1c was found to correlate with an increased risk of infection in patients undergoing spinal instrumentation (193). Hikata et al. found that patients with diabetes had a 16.7% chance of SSI, and those without diabetes had a 3.2% chance. Based on their findings, Hikata et al. recommend an HgbA1c of 7% or less before surgery (193). Smoking is associated with increased risk of SSI and delayed healing. One study by Sorensen et al. showed that SSI rates in smokers were 12 vs. 2% in non-smokers. The authors showed that, if smokers stopped smoking for 4 weeks before surgery, the SSI rates became equal to non-smokers (194). Opioid dose when initiating IDD has been shown to be an important factor as it relates to escalation of opioid dose after implant of an IDDS. Utilizing low opioid dosing may allow for limiting overall opioid dose, curbing opioid dose escalation, and achieving analgesia (88, 89). Type of pain may be taken into account when initiating treatment with IDD. While the 2017 PACC guidelines take note of type of pain (nociceptive and neuropathic), the authors did not specifically make recommendations for one type of pain vs. the other. Prior iterations of the PACC guidelines did break out treatment algorithms for nociceptive vs. neuropathic pain (195). At present, there is paucity of evidence to make specific recommendations for a particular IT medication in the treatment of neuropathic pain. There is no enough supportive evidence that IT opioids are, more or less, effective in treating neuropathic or nociceptive pain (107, 192, 195, 196).

The patient with cancer deserves a separate comment. With an ever-increasing survival rate, the prevalence of cancer pain is also increasing (197, 198). Although the vast majority of chronic cancer pain can be controlled with pharmacological therapies, many patients do not achieve adequate analgesia despite the availability of many regimens (199). Intrathecal drug delivery may provide an alternative route of administration for analgesics, which may improve pain relief and control of side effects (147, 199–204). The PACC recommendations (72) confirmed the role of intrathecal analgesia in the treatment of cancer pain with a high level of evidence (I) and a strong recommendation (Rank A). Likewise, the European Society of Medical Oncology (ESMO) (202), as the first time, recommends intrathecal analgesia for patients with cancer presenting with refractory pain and, especially, in those experiencing pain in multiple locations: head and neck, upper and lower extremities, and trunk, although it is more likely to be useful for pain below the diaphragm. Despite the accumulated evidence, the controversy and conflicting opinions continue, and it seems an indication that, unfortunately, still requires greater dissemination and general knowledge for the benefit of patients with cancer (204).

Pre-operative optimization and consideration of any patient condition that may impact outcomes is important. A potential delay upfront to optimize the patient may be necessary and avoid a potential loss of therapy down the line due to complication.

## Complications and Their Avoidance

Complications of IDD can be technical (e.g., IDDS malfunction) biological (e.g., infection) or other (e.g., granuloma). Complications can arise intra-operatively but most commonly occur post-operatively.

### Technical Complications

#### Intraoperative

Proper care begins in the pre-operative phase with appropriate patient selection, recognition, and management of pre-operative medication, optimization of the patient's medical status, and planning surgical placement, including a catheter entry site and pump placement location. Intra-operatively, meticulous adherence to proper surgical technique minimizes complications (205). Care must be taken to minimize risk of damage to the cord or conus with needle or catheter advancement being a good practice, according to the authors' own experience, to do it with the patient awake and communicative during the insertion of the needle and the catheter. The preferred positioning is in the lateral decubitus position for placement of the pump within the abdominal wall—although some use the prone position with placement of the pump in the buttock region. Proper intraoperative prep, drape, pre-operative antibiotics, double gloving, minimizing operating room traffic, and gentle manipulation of surgical tissues are important means for reducing the risk of perioperative surgical site infection. Proper hemostasis and creating a pocket just large enough to accommodate the pump are effective means of reducing the risk of hematoma and seroma formation, respectively. Placing a non-absorbable purse string suture around the catheter site may minimize the risk of CSF leak/hygroma. Adherence to published guidelines for perioperative use of anticoagulants is important for reducing the risk of bleeding (64, 206).

#### Post-Operative Complications

Maintenance of IDDS and troubleshooting complications are essential to managing implanted patients. The most common complications are catheter related, including catheter migration, occlusion, leak or fracture (207–209). Much less common are motor stalls in peristaltic pumps (207, 210, 211). Evaluation of suspected device malfunction begins with history, physical examination and device interrogation. If interrogation reveals pump malfunction, urgent replacement is indicated. If history, physical examination or device interrogation suggests system dysfunction, further action is needed, ranging from further investigation to urgent device revision/replacement, depending on the clinical scenario. Clinical investigation may include imaging, such as X-ray, CT scan, radionuclide scans, and catheter dye studies (208, 212). Skin erosion over the implant site often necessitates device removal, with special precautions for withdrawal, depending on intrathecal agents (213).

Surgical site infections (SSI) represent the most common biological complication of IDD. IDDS-related SSI can occur at either the catheter incision site or the pump site, with the latter being more common (214). As reviewed by the PACC guidelines, a number of factors are associated with a higher risk of SSI, including poorly controlled diabetes, anemia, smoking, immune suppression, cancer, cardiac disease, obesity, malnutrition, and active alcohol and drug abuse (64). Optimization of pre-operative status, including glycemic control with hemoglobin A1C <8% and smoking cessation for around 2 months prior to surgery, may curb the risk of infection. Screening by nasal swab for staphylococcus colonization has been recommended, and, if positive, appropriate treatment with topical mupirocin is indicated. Intraoperatively, washing the pump pocket with 20-ml vancomycin, 1 mg/ml along with pre-operative chlorhexidine prep, has been reportedly to markedly reduce SSI in pump exchange surgeries for intrathecal baclofen delivery (215). Placing an occlusive dressing has been recommended (64), and applying topical antibiotic may help reduce SSI (216). A detailed discussion of risk mitigations has been entertained by the PACC guidelines (64).

## Neuraxial Drug Safety

The direct application of therapeutics to the intrathecal space is potentially fraught for several reasons: (i) The therapeutics are often delivered in exceedingly high concentrations (e.g., morphine, 20 mg/ml; Lidocaine: 50 mg/ml); (ii) the local tissues are directly exposed to these concentrations: (iii) Given the relatively poor local redistribution secondary to the modest local flow, the local tissues may be exposed for an extended period, particularly if this delivery is by local infusion. All of these events are considered to potentially contribute to the adverse effects seen after intrathecal delivery. This section limits themselves to brief commentaries regarding pathologies, which have largely been identified secondary to neuraxial delivery of agents targeting analgesic processing.

## Intrathecal Catheter Tip Granulomas

In 1991, North and colleagues reported a case of paraplegia induced by a dorsal spinal mass at T10-11 adjacent to an implanted intrathecal catheter (217). The patient had a history of intractable chronic low back pain, culminating in an implant of an intrathecal drug delivery device 14 months prior to presentation. The patient was titrated up to receiving 100-mg intrathecal morphine and felt better initially. However, 2 months after the implant, the patient developed progressive paraparesis and, within a month later, was paraplegic. Upon presentation, the patient had flaccid paraplegia, was insensate to touch and areflexic below T10, and had developed decubitus ulcers. CT myelography revealed a soft tissue mass at the catheter tip. Surgical excision revealed a large sterile inflammatory mass. The formation of a sterile fibrotic mass at the catheter tip (granuloma) is usually associated with high dose and concentration of IT opiates; and most is commonly seen with morphine and hydromorphone (see below) (218–224). The incidence of masses with IT fentanyl appeared to be minimal (225). Preclinical work characterized these intrathecal granuloma masses as being a proliferation of fibroblasts embedded in a collagen matrix, arising from the adjacent dura-arachnoid mater (226). In this series of studies, the intrathecal pericatheter mass was shown to have several defining properties. (i) Infusion of opioids, such as morphine or hydromorphone resulted in intrathecal catheter tip granuloma (ICTG) formation. On the other hand, an ICTG was not observed with intrathecal infusion of saline or synthetic opioids fentanyl, alfentanil, and several opioid peptides [D-Ala^2^, N-MePhe^4^, Gly-ol]-enkephalin (DAMGO) (83). (ii) The masses were dependent upon high infused concentrations (e.g., vs. total dose delivered) and developed proximal tothe catheter tip where the solute concentrations to which the tissue was exposed were, indeed, highest (84). (iii) The lack of a conventional opioid structure activity relationship (e.g., effects produced by morphine but not fentanyl) along with failure to reverse or prevent the onset of the effect with an opiate antagonist suggested that the effects were not mediated by an opiate receptor. (iv) It had been noted that the opiates causing mast cell degranulation (e.g., morphine) led to mass formation, whereas those that did not (e.g., fentanyl) degranulate meningeal mast cells (227) did not lead to such masses (83). Support for this mast cell linkage was suggested by the observation that administration of subcutaneous or intrathecal cromolyn, a mast cell stabilizer, prevented granuloma formation (226). This suggested that the mechanism for ICTG formation was mediated by meningeal mast cell degranulation in response to the opiate. (v) It was shown that these opioid agonists acted to degranulate mast cells by their activation of Mas receptor G protein-coupled receptors (e.g., MrgprB2-mouse and MrgprX2-human) (86). Work with the mu opioid agonists that did not activate MRGs was found not to produce mast cell degranulation, fibroblast proliferation, and did not produce masses (85, 228). Subsequent work with MRG mutant mice confirmed these findings (219). Future development of such non-MRG-activating molecules, given their absence of a risk of mass formation, may hold promise for future analgesics.

Clinically, multiple case reports and series described ICTG formation predominantly in response to morphine and hydromorphone with an incidence approaching 9% in larger case series (220, 229, 230). Outside of two case reports with extremely high daily doses of fentanyl (2.7 and 8.9 mg/day), there have been no reports of pericatherer masses with intrathecal fentanyl. Overall, ICTG's have been reported with a number of intrathecal agents and may reflect an interaction between high concentrations of the intrathecal agent and limited local cerebrospinal fluid flow, resulting in sensitization of dural mast cells to release histamine and trigger a cascade leading to mass formation. The diagnostic modality of choice is magnetic resonance imaging with and without the use of gadolinium, with special care to acknowledge the catheter tip artifact. Management is typically conservative with expeditious wean of intrathecal opioids and replacement with saline, followed by revision of the intrathecal catheter to a different location and the use of non-granuloma, inducing medication if the patient desires to continue to use IDD. Surgical management is reserved to limited cases with larger lesions and progressive acute neurological deterioration (175). Recent experimental data have suggested that multiple daily bolus dosing results in lesser granuloma formation compared to continuous intrathecal infusion (175). The use of multiple small boluses vs. continuous low rates of infusion is believed to lower the mass formation incidence as the bolus results in increased dilution of the high solute concentration. This is discussed further below.

## NMDA Antagonists

Intrathecal delivery of agents, such as ketamine, has been employed in the management of severe pain states in humans (231, 232). Intrathecal infusions of several NMDA antagonists in large animal models have revealed a general parenchymal pathology. It should be noted that the concentrations required for analgesia were typically not identified, and, thus, it is not known if the doses markedly exceed those which were required for a therapeutic effect in these animal models (233). In a single human neuropathic pain case, examination of spinal cord and nerve roots after neuraxial administration of S (+)-ketamine (and numerous other products) revealed histological abnormalities, including central chromatolysis, nerve cell shrinkage, neuronophagia, microglial upregulation, and gliosis (234). In a second finding, studies with exposure to ketamine in the neonatal rat were shown to produce a concentration-dependent analgesia, but, at comparable doses, an increase in dorsal horn apoptosis was identified (235). The origins of this pathology have not been identified.

## Local Anesthetics

The early studies with intrathecal local anesthetics in the 1900s up through today have emphasized that delivery of molecules with a local anesthetic profile led to an increased incidence of radiculopathies, a finding in concert with preclinical studies of drugs placed near nerves (236, 237). Several local anesthetics (lidocaine, bupivacaine, 2 Chloroprocaine) induce demyelination and radiculopathy in several species, including rats, rabbits, dogs, and humans. Extensive work has emphasized that these effects were concentration dependent (237–240). The mechanism of this toxicity is controversial. This neurotoxicity is expressed in the sensory neuron and results from an action secondary to an anesthetic-induced increase in intracellular Ca++ (241). An additional mechanism has been proposed, including upregulation of CaMKIIβ along with an increase in Cav3.2 and Cav3.3, and activation of apoptotic signaling (242).

## Adenovirus Transfection

Observations of vector-treated animals (mice, primate) have shown moderate asymptomatic degeneration of dorsal root ganglia neurons (a “ganglionitis”) and associated axons (154, 243), but not in cats and dogs (244, 245). In primates, a mononuclear pleocytosis in the cerebrospinal fluid has been observed (243, 246). Where examined, these effects appear to be titer dependent, but independent of an immune response. Pertinent study variables aside from the viral vector itself are titer and route of delivery.

## Organizing Principles of Neuraxial Pathology

i) Our experience is that the safety of a product that has been well-studied by different systemic routes should not be taken as an indication of intrinsic neuraxial safety. The result with intrathecal opiates, local anesthetics, and NMDA antagonists speaks directly to the refutation of that assertion. This emphasizes the importance of systematic preclinical evaluation of the safety in validated animal models that address the limits of the concentrations and exposure profile to be employed in humans. Changes in concentration or formulation must be considered as non-trivial. To paraphrase Paracelsus: There are no safe drugs – only safe doses (concentrations). This also applies to biologics where the designation of two products as equivalent raises the issues of how to define the equivalency of two “biosimilars” (247). The pathology defined for one cannot, out of hand, be considered as evidence for the safety of a similarly targeted but differently constructed therapeutic. (ii) It is worthwhile noting that the single common factor linking the diverse pathology phenotypes is that they reflect the role of local concentration in the evolution of the various pathologies. This has practical implications. Here, we note that, for a given concentration, an increase in the injected volume (increasing dose) leads to a greater spread of the solute, but the maximum concentration at the site of delivery is essentially the same, although that concentrations spread over a greater distance. So, while total volume increases the total system exposure, it does not alter the local concentration proximal to the catheter. On the other hand, keeping the volume of delivery constant and increasing concentration, indeed, increases dos, but, more importantly, the exposure of the local tissue is increased. So, to the degree that local pathology is the concern, the assessment of that risk is related to the concentrations applied and not the total dose (25).

## Summary

Intrathecal therapy is an important strategy to treat patients with chronic pain and has its own entity to define the specific indications that allow the selection of the ideal patient for therapy. With the new imaging technologies and, especially, computerized simulation, we can know the best interaction of the anatomy and physiology of the CNS with IDD therapy. Cerebrospinal fluid (CSF) dynamics, CSF elimination pathways, and the location and volume of the injected bolus can predict regional exposure of CNS tissue to molecules with variable chemical properties and thus achieve the best therapy outcome. From basic research, very active, we conclude that therapy evolves, providing a perspective that provides new strategies and innovations. Although complications can occur, as in other therapies, the main problems do not arise from the device itself, but mainly with inadequate patient control, inflammatory mass (e.g., high doses and concentrations of opioids), wound healing, dosing errors (e.g., drug concentration and pump programming), pump refills or refills (e.g., pocket refills), and interaction with concomitant systemic medications (e.g., opioids and benzodiazepines). Therefore, they can be prevented through proper training of the doctor, the implementation of best practices and experience, resulting in best practice and results for the management of patients with severe chronic pain.

## Author Contributions

JA, SH, and TY conceived and planned the article content and its sections. JA, SH, CP, ML, MR, CA-S, RR-H, MH, and TY, carried out the search of information for each specific section and contributed in writing the manuscript. All authors provided critical feedback and helped shape the analysis and final preparation of figures and text of manuscript.

## Funding

TY received funding from R01 NS102432 and R01NS099338.

## Conflict of Interest

CP is a consultant for Medtronic (honoraria for scientific advisory board and consultation fees). TY holds consulting agreements with Medtronic and Sorrento and is on the SAB for Raft and Navega. TY and MH hold a patent for an intrathecal catheter described herein. The remaining authors declare that the research was conducted in the absence of any commercial or financial relationships that could be construed as a potential conflict of interest.

## Publisher's Note

All claims expressed in this article are solely those of the authors and do not necessarily represent those of their affiliated organizations, or those of the publisher, the editors and the reviewers. Any product that may be evaluated in this article, or claim that may be made by its manufacturer, is not guaranteed or endorsed by the publisher.
