# Corrigendum: Intrathecal drug delivery: Advances and applications in the management of chronic pain patient

**DOI:** 10.3389/fpain.2023.1190014

**Published:** 2023-04-11

**Authors:** Jose De Andres, Salim Hayek, Christophe Perruchoud, Melinda M. Lawrence, Miguel Angel Reina, Carmen De Andres-Serrano, Ruben Rubio-Haro, Mathew Hunt, Tony L. Yaksh

**Affiliations:** ^1^ Surgical Specialties Department, Valencia University Medical School, Valencia, Spain; ^2^ Anesthesia Critical Care and Pain Management Department, Valencia, Spain; ^3^ Department of Anesthesiology, University Hospitals Cleveland Medical Center, Cleveland, OH, United States; ^4^ Pain Center and Department of Anesthesia, La Tour Hospital, Geneva, Switzerland; ^5^ Lausanne University Hospital and University of Lausanne, Lausanne, Switzerland; ^6^ Department of Anesthesiology, Montepríncipe University Hospital, Madrid, Spain; ^7^ CEU-San-Pablo University School of Medicine, Madrid, Spain; ^8^ Department of Anesthesiology, University of Florida College of Medicine, Gainesville, FL, United States; ^9^ Facultad de Ciencias de la Salud Universidad Francisco de Vitoria, Madrid, Spain; ^10^ Multidisciplinary Pain Clinic, Vithas Virgen del Consuelo Hospital, Valencia, Spain; ^11^ Anesthesia and Pain Management Department, Provincial Hospital, Castellon, Spain; ^12^ Multidisciplinary Pain Clinic, Vithas Virgen del Consuelo Hospital, Valencia, Spain; ^13^ Department of Physiology, Karolinska Institute, Stockholm, Sweden; ^14^ Departments of Anesthesiology and Pharmacology, University of California, San Diego, San Diego, CA, United States

**Keywords:** antisense, intrathecal, neuromodulation, chronic pain, implantable drug delivery system (IDDS)

A Corrigendum on Intrathecal drug delivery: Advances and applications in the management of chronic pain patient By De Andres J, Hayek S, Perruchoud Ch, Lawrence MM, Reina MA, De Andres-Serrano C, Rubio-Haro R, Hunt M and Yaksh TL. (2022) Front. Pain Res. 3:900566 doi: 10.3389/fpain.2022.900566

In the published article, there was an error in the figure and legend of Figure 9. In the incorrect figure, the two columns of recording are switched. The *T* = 15 s image should be *T* = 240 s and the *T* = 240 image should be *T* = 15 s. The corrected figure is the same except the images switched to correct for the mislabeled timing. The figure legend is now as follows.

“FIGURE 9 Two-dimensional diffusion chamber with a catheter having 20 microports. Images taken at 15 s (top, left) and 240 s (bottom, left), following bolus delivery of 2.6 µl as a bolus at time 0. The image on the right of the *T* = 15 s image shows an enlargement of the high velocity stream exiting the catheter at two of the exit valves, with the characteristic mushroom head where dye laden solute encounters the local dye-free fluid phase. Note the even distribution of dye from proximal (pump) to distal over the 6-cm catheter distance (T. L. Yaksh)”

**Figure:**
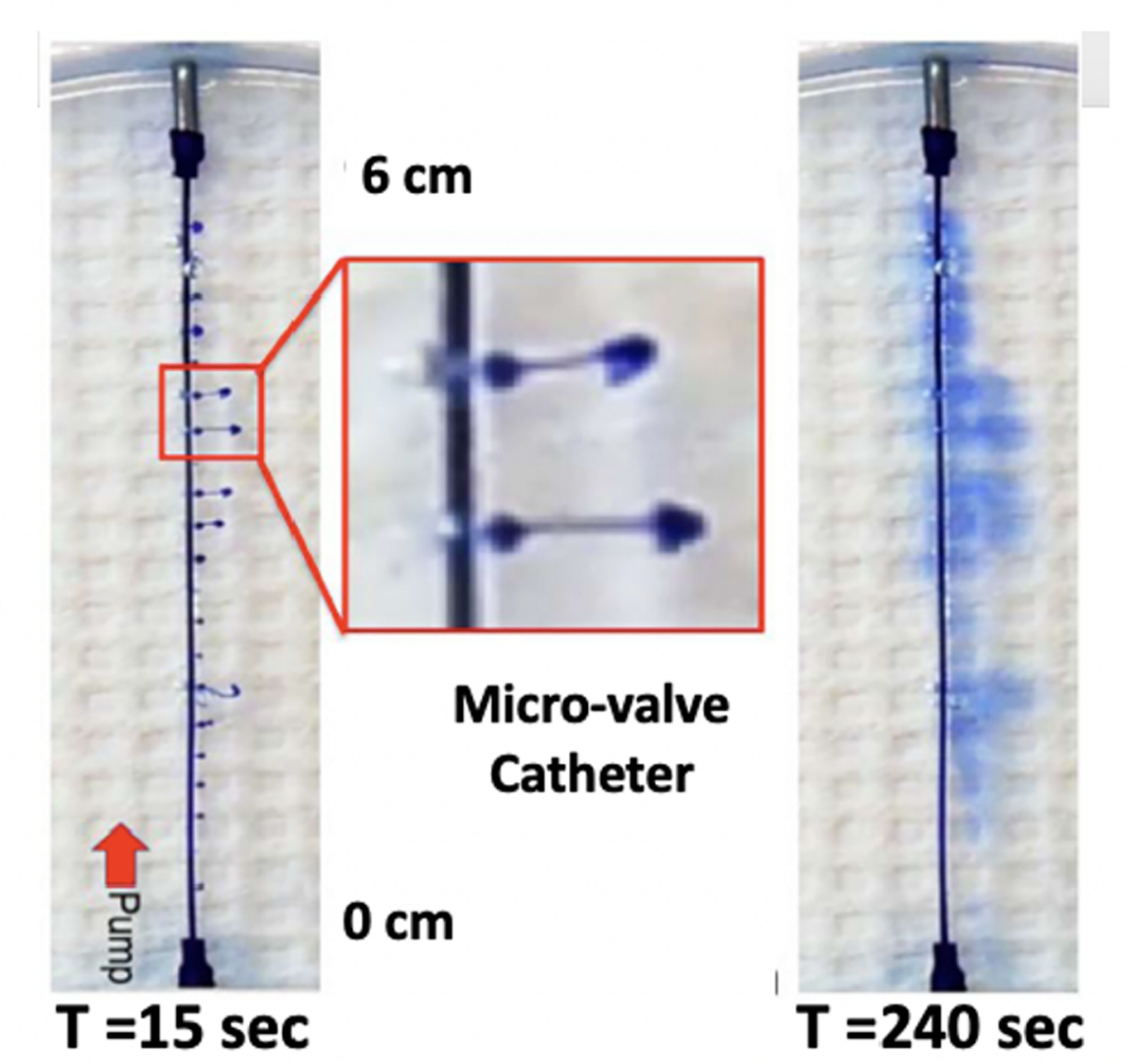


The authors apologize for this error and state that this does not change the scientific conclusions of the article in any way. The original article has been updated.

